# Fe_4_S_4_ Cubane Type Cluster Immobilized on a Graphene Support: A High Performance H_2_ Evolution Catalysis in Acidic Water

**DOI:** 10.1038/s41598-017-17121-7

**Published:** 2017-12-05

**Authors:** Ameerunisha Begum, Aasif Hassan Sheikh, Golam Moula, Sabyasachi Sarkar

**Affiliations:** 1 Department of Chemistry, Faculty of Science, Jamia Hamdard University, New Delhi, 110062 India; 2 Nanoscience and Synthetic Leaf Laboratory at Downing Hall, Center for Healthcare Science and Technology, Indian Institute of Engineering Science and Technology, Botanic Garden, Howrah, 711103 West Bengal India

## Abstract

The development of alternate catalysts that utilize non-precious metal based electrode materials such as the first row transition metal complexes is an important goal for economic fuel cell design. In this direction, a new Fe_4_S_4_ cubane type cluster, [PPh_4_]_2_[Fe_4_S_4_(DMET)_4_] (**1**) (DMET = cis-1,2-dicarbomethoxyethylene dithiolate) and its composite with functionalized graphene, (**1**@graphene) have been synthesized and characterized. The presence of nanocrystalline structures on graphene matrix in TEM and SEM images of **1**@graphene indicate that the cluster (**1**) has been immobilized. The composite, **1**@graphene evolves H_2_ gas from p-toluene sulfonic acid (TsOH) in a mixture of H_2_O and CH_3_CN under ambient conditions with a significant turnover number of 3200. **1**@graphene electro-catalyzes H_2_ evolution at E_p_, −1.2 V with remarkable throughput, catalytic efficiency and stability in only H_2_O or in only CH_3_CN. The Fe_4_S_4_ cluster (**1**) alone electro-catalyzes hydrogen evolution at E_p_, −0.75 V from TsOH in CH_3_CN. The X-ray crystal structure of the Fe_4_S_4_ cluster (**1**) (λ_max_, CH_2_Cl_2_, 823 nm; ε, 2200 mol^−1^ cm^−1^) shows that it is dianionic with a cumulative oxidation state of +2.5 for the iron centers and short C-S bond distances (ca., 1.712 Å & 1.727 Å) indicating the presence of sulfur based radicals.

## Introduction

**Figure 1:**
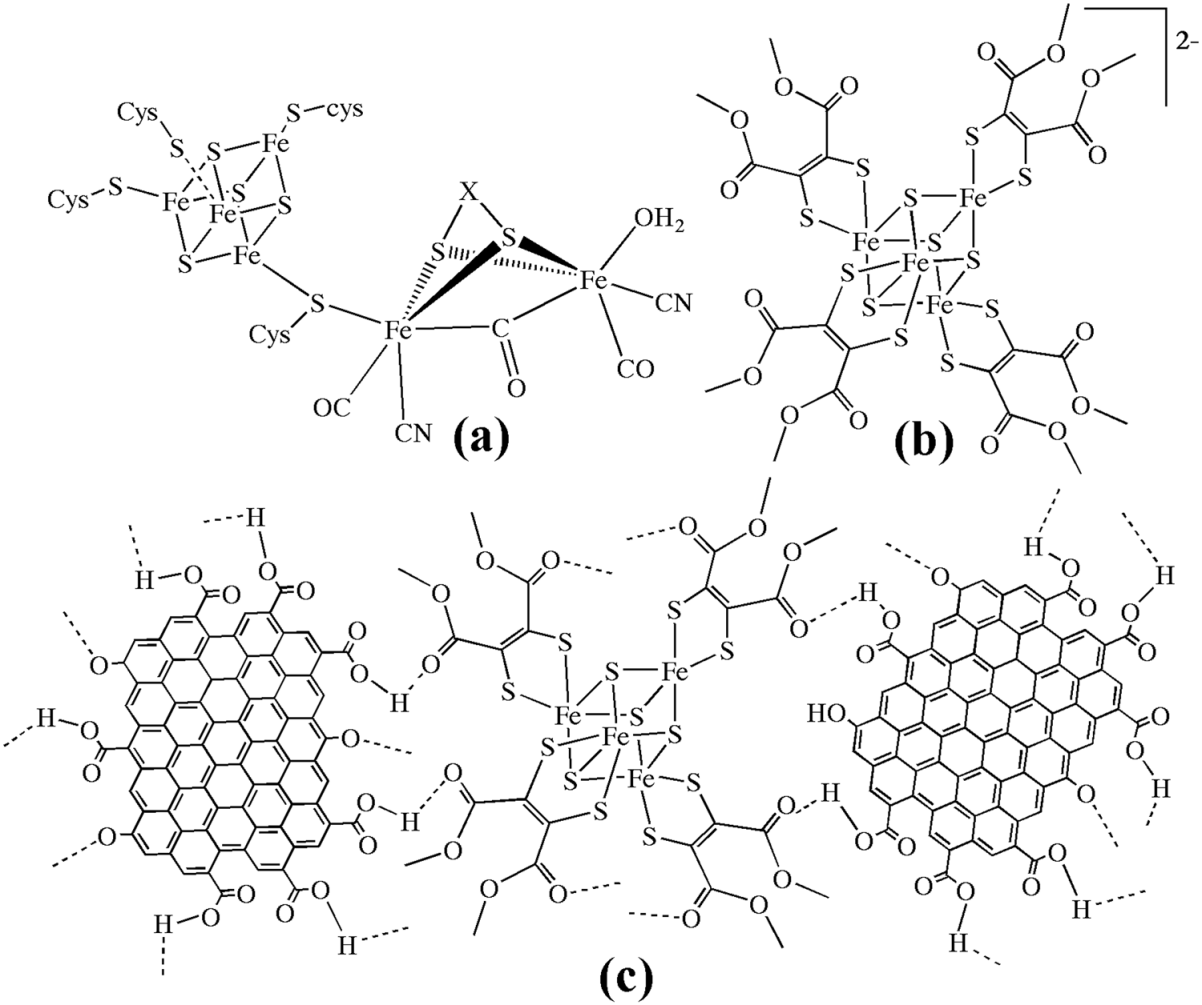
Structures of (**a**) H-cluster in H_2_ evolving *Clostridium pasteurianum* ^1^, (**b**) anionic part of the cluster (**1**), (**c**) H-bonding interactions in **1**@graphene.

The presence of the dimetallic iron and heterodimetallic iron-nickel centers at the active sites of the hydrogenases has evoked the researchers to search for a non-platinum catalyst material for proton reduction and H_2_ splitting. The nanocomposites of hydrogenase mimics with large surface area and versatile electronic behavior are expected to catalyze reduction of protons to hydrogen gas at lower electro-potentials than platinum based electrode materials^1–5^. The three known classes of hydrogenases, [NiFe]−, [FeFe]− and FeS−cluster free hydrogenases contain iron at their active site which is coordinated by thiolates, CO, CN^−^ or a light sensitive cofactor (Fig. 1 a)^6–13^. Electro-catalytic H_2_ generation involves reduction of protons to H_2_ gas at lower reduction potentials towards E_p_, 0.0 V under a catalyst that can performance-wise replace the platinum electrode (i.e., −0.413 V at pH 7.0)^14–21^. Several electro-catalysts for hydrogen evolution have been reported including a series of multinuclear iron sulfur complexes with benzene tetrathiolate bridges, iron carbonyl clusters, cobalt-dithiolene, metallo-porphyrins, low-valent transition metal complexes, diiron dithiolates, multinuclear Fe-S cluster with a Fe_cubane_(μ-SR)Fe_subsite_ linkage, molybdenum-sulfur dimers, cobalt glyoximes, substituted iron glyoximes, carbon nanotube grafted nickel bisdiphosphine complexes, and mononuclear iron(II) polypyridyl complexes^22–29^.

Nickel(II)− and iron(III) dithiolenes have been reported which electro-catalyze H_2_ evolution at potentials comparable to that of platinum disc electrode^30–32^. Recently the research on light driven H_2_ production has gained momentum. Biomimetic chalcogels incorporating Fe_4_S_4_ cubane type clusters linked with (Sn_2_S_12_)^4−^ blocks and iron dithiolene complexes have been reported as catalysts for light driven H_2_ production from water and as solar fuel catalysts^33,34^. The Fe_4_S_4_ cubane type cluster take part in many biological electron transfer processes which are also coupled with several other enzymatic reactions. The 6H^+^-6e^−^ reduction of N_2_ to NH_3_ by Fe_4_S_4_ cubane type cluster alone has been reported by van Tamelen *et al*.^35^. Hence, we are interested to find out the efficiency of Fe_4_S_4_ cubane type cluster alone as electrochemical hydrogen evolution catalysts. Since the Fe_4_S_4_ cubane type clusters are embedded in protein matrix in the biological active site, the stability and catalytic efficiency of the Fe_4_S_4_ clusters might be enhanced if they can be embedded or immobilized on a graphene support. Two dimensional nanomaterials of various compositions including graphene have been studied as catalysts in electrochemical reactions such as hydrogen evolution reaction (HER), oxygen reduction reaction (ORR), oxygen evolution reaction (OER) and carbon dioxide reduction^36^. Recently 2D nanomaterials including functionalized graphene, their hybrids have gained interests in electrochemical energy conversion and storage devices^37^. Defects existing in the 2D nanomaterials play an important role in tailoring of optical and electric properties. Functionalized graphene obtained by oxidation contains –COOH, –C=O, and –OH functional groups which can be used to immobilize metal ions and organic groups. Several graphene-photocatalyst composites have been developed recently for HER^38^. Graphene as a matrix would enhance the catalytic performance of the electrocatalysts embedded on it due to its large surface area, excellent conductivity and indefinite durability. Graphene oxide gets folded to close like fisted form and entraps a large molecule such as tetraphenylporphyrin (TPP)^39^. Several graphene based hybrid electrocatalysts as anodic and cathodic fuel cell electrode materials have been reviewed^40^. Here in we report a new Fe_4_S_4_ cubane type cluster, [PPh_4_]_2_[Fe_4_(μ_3_–S)_4_(DMET)_4_] (**1**, Fig. 1 b) and a functionalized graphene composite, **1**@graphene (Fig. 1 c) which react with protons and evolve H_2_ gas in a mixture of CH_3_CN and water under ambient conditions and electrochemically at E_p_, −1.21 V in CH_3_CN or in H_2_O.

## Results and Discussion

### Synthesis and X-ray structural data of the Fe_4_S_4_ cubane type cluster, [PPh_4_]_2_[Fe_4_(μ_3_-S)_4_(DMET)_4_] (1)

**Figure 2:**
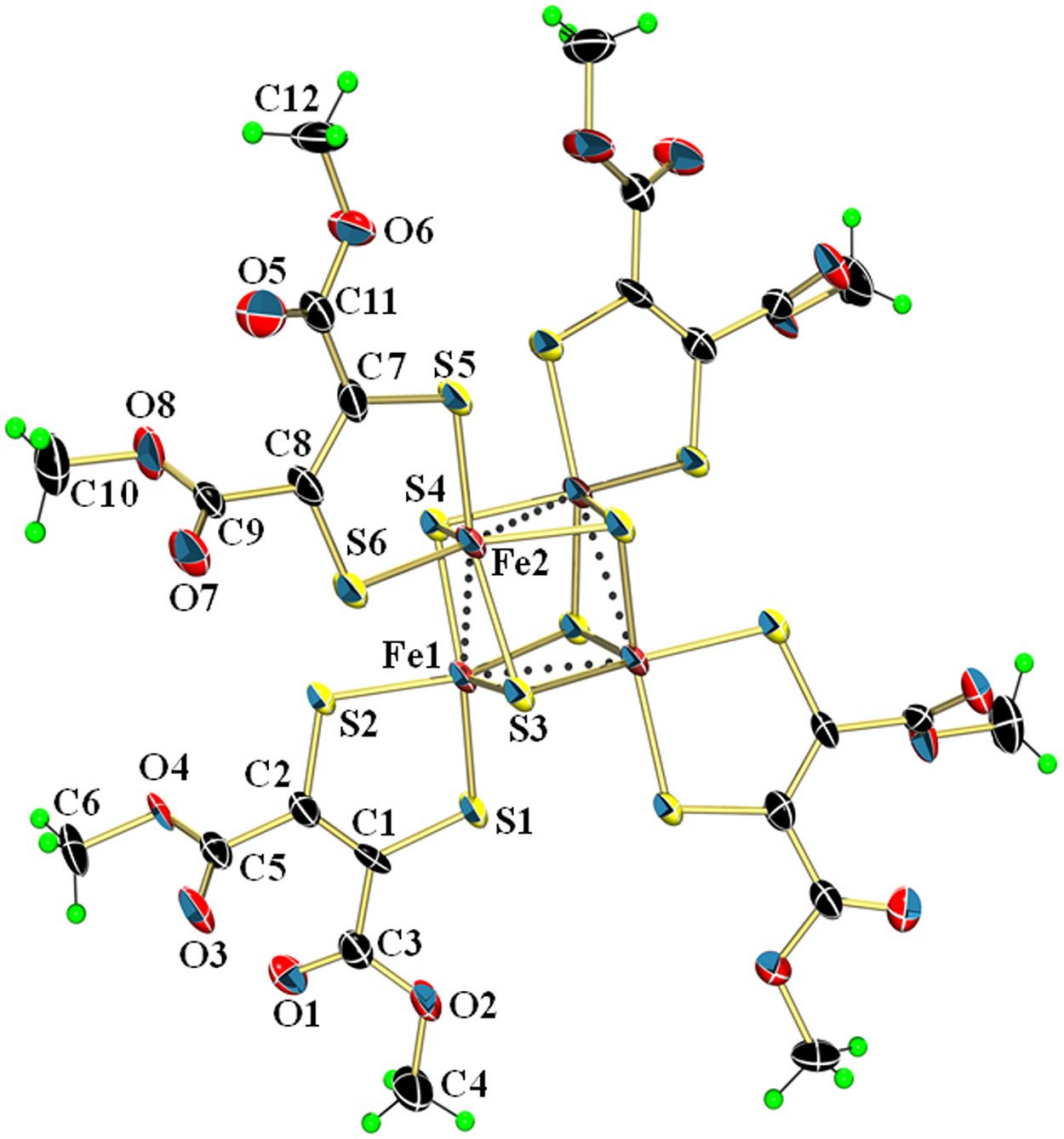
Perspective view of the anionic part of the cluster (**1**) in 50% probability thermal ellipsoids (hydrogen green; carbon, black; oxygen, red; sulfur, yellow; iron, brown). Selected bond lengths (Å): Fe(1)-S(1), 2.1830(15); Fe(1)-S(2), 2.2009(15); Fe(1)-S(3), 2.2429(15); Fe(1)-S(4), 2.1574(15); Fe(2)-S(3), 2.1563(15); Fe(2)-S(4), 2.2393(15); Fe(2)-S(5), 2.2037(15); Fe(2)-S(6), 2.1918(15); Fe(1)-Fe(2), 2.6947(11); Fe(2)-Fe(1), 2.7226(11); S(2)-C(4), 1.712(5); S(6)-C(10), 1.727(5).

The cluster (**1**) was prepared by the reaction of iron(II) polysulfide, [PPh_4_]_4_[Fe_2_^II^S_12_] with dimethylacetylene dicarboxylate (DMAD) and lithium sulfide (Li_2_S) in CH_3_CN under Schlenk conditions. The X-ray structure determination of (**1**) indicates the presence of dianionic, [Fe_4_(μ_3_-S_4_)(DMET)]^2−^ cluster and [PPh_4_] cations in 1:2 ratio. The ORTEP view of the anionic part of the cluster (**1**) is shown in Fig. 2 which confirms the formation of Fe_4_S_4_ core with four bidentate DMET ligands coordinated to iron. The geometry around each iron atom is square pyramidal formed by three inorganic sulfurs (S^2−^) and two thiolates of the DMET ligand. The Fe–S bond lengths are similar at all iron centers, (Fe(1) and Fe(2)) and in the range 2.156 Å–2.250 Å. This is in contrast to the longer Fe-S bond distances (2.246 Å–2.383 Å) in the tetra anionic, super reduced cluster, [NBu_4_]_4_[Fe_3_^III^Fe^II^(μ_3_-S)_4_(mnt)_3_^6−^(mnt)^1−·^]^4−·^ (**2**) (mnt = maleonitrile dithiolate)^32^. The Fe…Fe separations (Fe–Fe, 2.695 Å and 2.722 Å) are remarkably shorter in the cluster (**1**) in contrast to those observed in (**2**) (Fe-Fe, 2.843 Å–3.123 Å) and four of the C–S bond distances (S(2)–C(2) & S(2A)–C(2A), 1.718 Å and S(6)–C(8) & S(6A)–C(8A), 1.721 Å) are considerably shorter in (**1**) indicating oxidation of the dithiolene ligands to their monoanionic form containing sulfur based radicals. The lattice packing in the crystals of (**1**) indicates that the anionic Fe_4_S_4_ cluster is fully covered by a group four [PPh_4_] cations with considerably short S^2−^(cluster)…H–C(phenyl) (2.91 Å and 3.45 Å) and O(cluster)…H–C(phenyl), (2.43 Å). All of the four S^2−^ in the anionic part are involved in short contacts with C-H of the PPh_4_ cations. These interactions might provide stability to the cluster skeleton and are reminiscent of the intermolecular interactions observed in native proteins.

**Figure 3:**
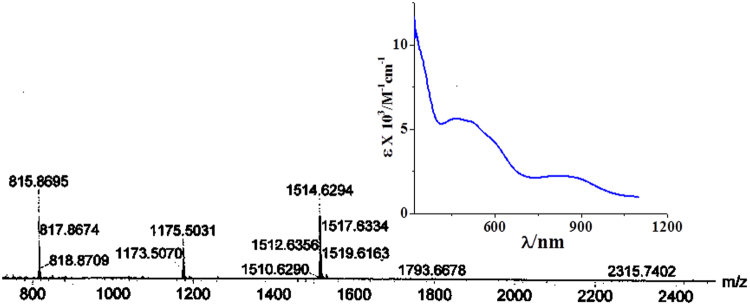
ESI-MS (negative) of the cluster (**1**) in CH_3_CN. (Inset: UV-VIS spectrum of (1) in CH_2_Cl_2_.

The dianionic 1,2-dithiolenes coordinated to metal complexes are known to undergo oxidation resulting in monoanionic ligands with S–based radicals^41–47^. The considerably shorter C−S bond distances in a previously reported [Fe_4_(μ-S)_4_(S_2_C_2_(CF_3_)_2_)_4_]^2−^ dianion and in cluster (**1**) indicate that the four monoanionic dithiolates provide only four negative charges to the cluster whereas the four inorganic sulfurs (S^2−^) provide eight negative charges making up a total of 12 negative charges which are satisfied by the four iron ions and two [AsPh_4_]/[PPh_4_] cations^48,49^. This confirms that the four iron centers are in a comprehensive oxidation state of +2.5 and the four dithiolene ligands have been oxidized to form monoanionic ligands with four S-based radicals. The appearance of a peak at m/z, 1514 in electro spray ionization mass spectrum (Fig. 3) indicates the stability of {[PPh_4_][Fe_4_(μ_3_–S)_4_(DMET)_4_]}^−^ complex ion in CH_3_CN. The complex (**1**) is EPR silent both in the solid state and in CH_2_Cl_2_ solution. The diamagnetic nature of the cluster (**1**) is understood from the room temperature magnetic moment (μ_eff_) of <1 μ_B_. The presence of a band around λ, 823 nm (ε, 2200 M^−1^cm^−1^) in the electronic spectrum of (**1**) in CH_2_Cl_2_ (Fig. 3) can be assigned to an intervalence-charge transfer (IVCT) band which is observed in sulphur based radical containing complexes. This indicates the presence of S–based radicals and since the complex (**1**) is diamagnetic, the S-based radicals are expected to be coupled.

### Synthesis and characterization of the composite, 1@graphene

**Figure 4:**
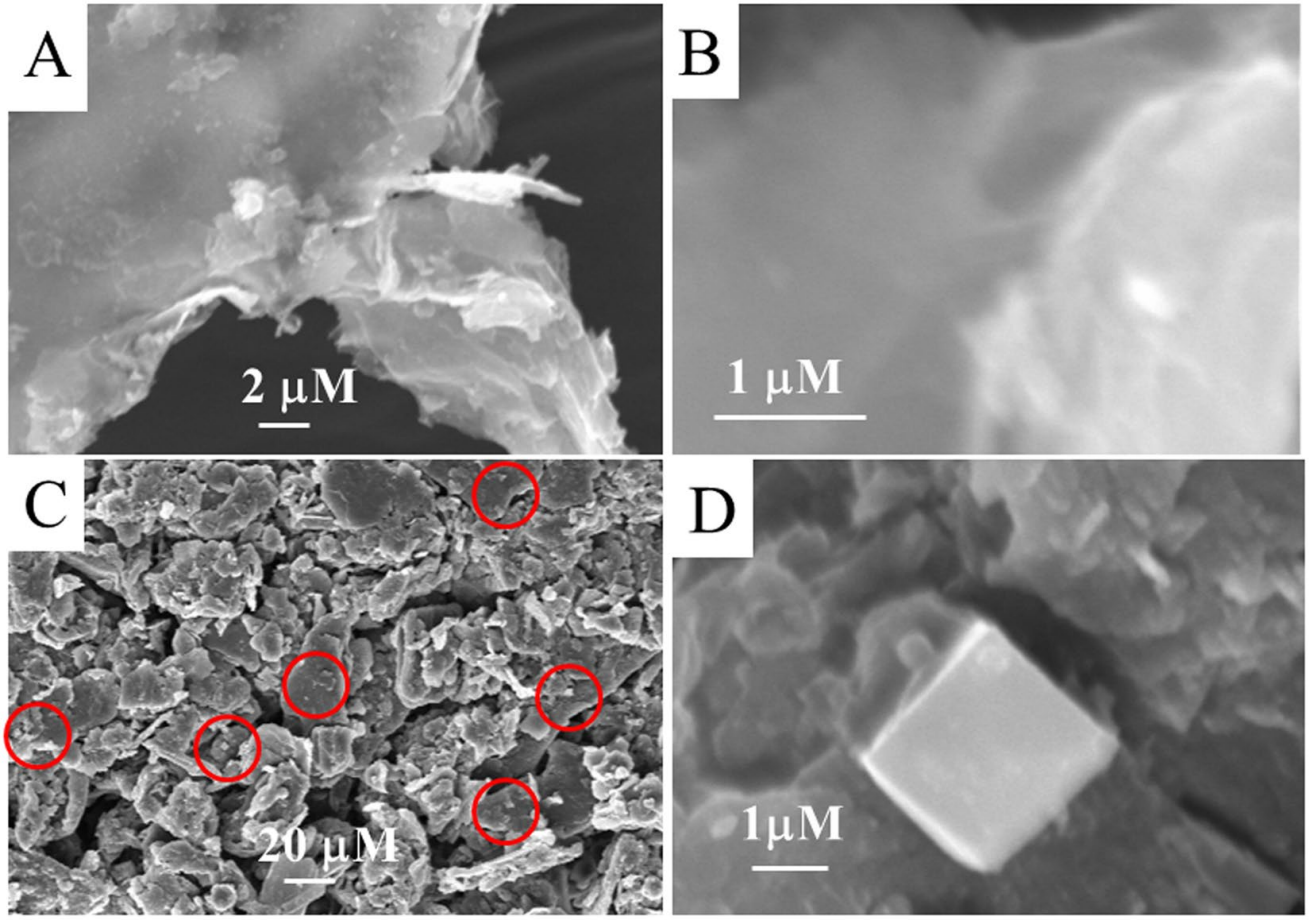
(**A** and **B**) SEM images of the functionalized graphene at low and higher magnifications on aluminium matrix. (**C** and **D**) SEM images of 1@graphene at low and higher magnifications on aluminium matrix. C, red circled area is magnified in D displaying the Fe_4_S_4_ cubane type cluster, 1 embedded in a bed of functionalized graphene.

**Figure 5:**
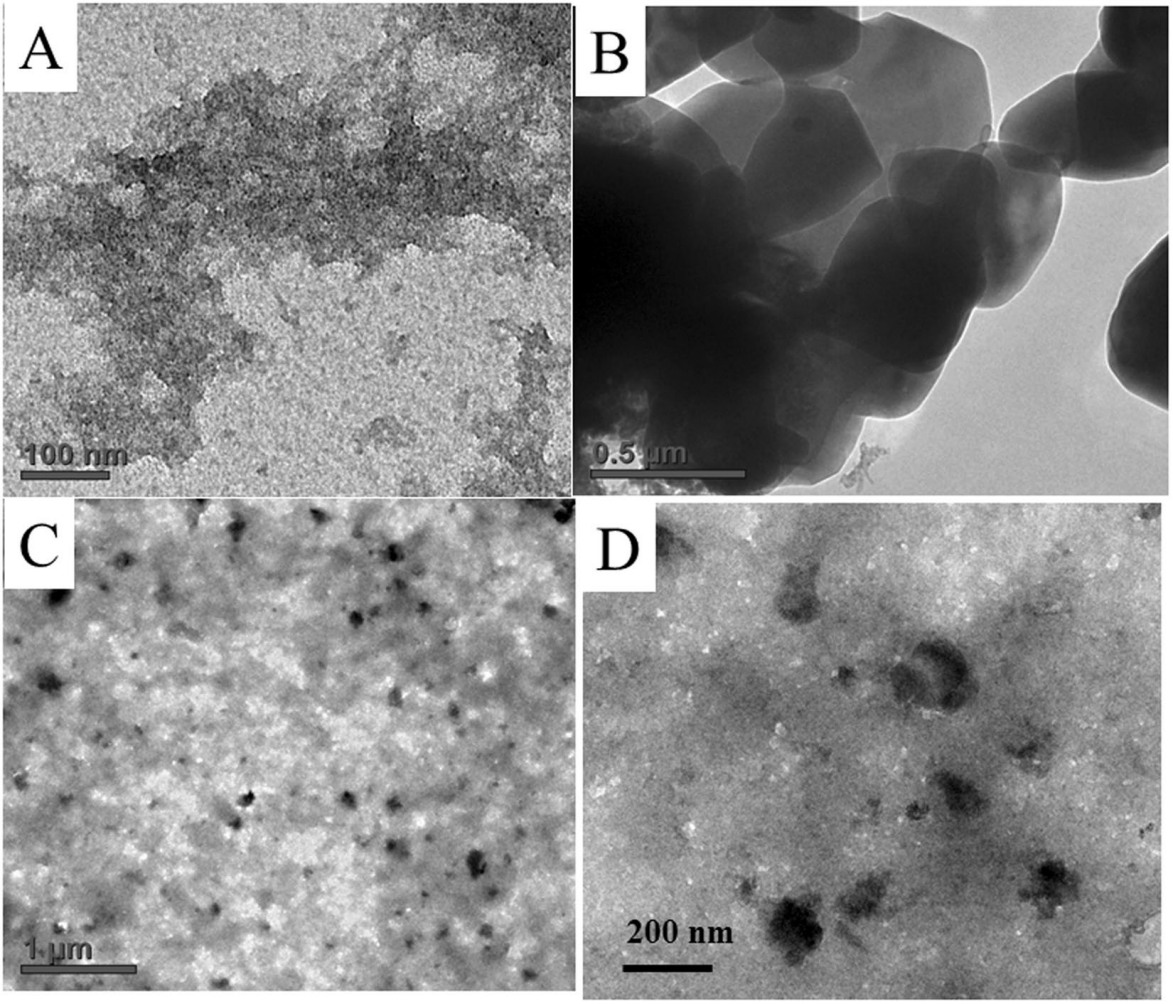
HR TEM images of f-graphene, Fe_4_S_4_ cluster (**1**) and **1**@graphene in CH_3_CN-H_2_O on carbon coated copper grids. (**A**) functionalized graphene. (**B**) Fe_4_S_4_ cluster (1). (**C** and **D**) **1**@graphene at low and high magnifications.

The composite, **1**@graphene was prepared by the ultrasonication of Fe_4_S_4_ cubane type cluster, (**1**) and functionalized graphene for 10 h in CH_3_CN-H2O under an argon atmosphere. It was then evapourated to dryness and the black residue was labelled as **1**@graphene. The IR spectral peaks of **1**@graphene show significant shifts to lower frequencies compared with the Fe_4_S_4_ cluster (**1**). The doublet at 1718 cm^−1^ and 1690 cm^−1^ observed in (**1**) due to –COO stretching of dithiolenes is shifted to 1616 cm^−1^ and 1610 cm^−1^ in **1**@graphene and the doublet is also broadened (Supplementary information). This indicates the existence of the H-bonding interactions with –COOH of the f-graphene as shown in Fig. 1 c. Furthermore, in **1**@graphene, a broad band is observed at 3324 cm^−1^ indicating the presence of hydrogen bonded –OH groups. Functionalized graphene and the composite, **1**@graphene were further analyzed by scanning electron microscopy (SEM) and results are displayed in Fig. 4. The sample containing functionalized graphene displayed transparent and thin filmy structures as shown in the Fig. 4A and B. The sample containing **1**@graphene diaplayed a mixture of graphene and the Fe_4_S_4_ cubane type cluster (**1**) as shown in Fig. 4C (low magnification) and Fig. 4D (higher magnification). The red circled area in Fig. 4C has been magnified and shown in Fig. 4D which indicates that a cubic structure is embedded in a bed of transparent filmy structure. The functionalized graphene, Fe_4_S_4_ cluster (**1**) and the composite **1**@graphene were dispersed in H_2_O-CH_3_CN, deposited onto carbon coated copper grids and analyzed by HR TEM. The results are displayed in Fig. 5 which indicates that the functionalized graphene solidifies as a film whereas the Fe_4_S_4_ cluster recrystallize as nanocrystals. The TEM images of **1**@graphene displayed in Fig. 5C and D indicated the presence of dark crystals/particles of Fe_4_S_4_ cluster on the graphene matrix. The Fe_4_S_4_ crystals can be immobilized on a functionalized graphene matrix through H-bonding interactions with the –COOH, -OH functional groups which has also been corroborated by IR spectral studies as described above. Because the Fe_4_S_4_ cubane type clusters crystallize in the form of nanocubes and microcubes as confirmed by our unpublished results on the cluster, [NBu_4_]_4_[Fe_3_^III^Fe^II^(μ_3_–S)_4_(mnt)_3_^6−^(mnt)^1−·^]^4−·^ (**2**) which displays nanocubes on recrystallization from CH_3_CN on a brass matrix and as nanotriangles on recrystallization from ethanol on a brass matrix. The EDX analysis of these nanocubes and nanotriangles confirmed the presence of carbon, iron, sulphur, phosphorous in the expected ranges as described in our unpublished results.

### Cyclic voltammetric data and proton reduction

**Figure 6:**
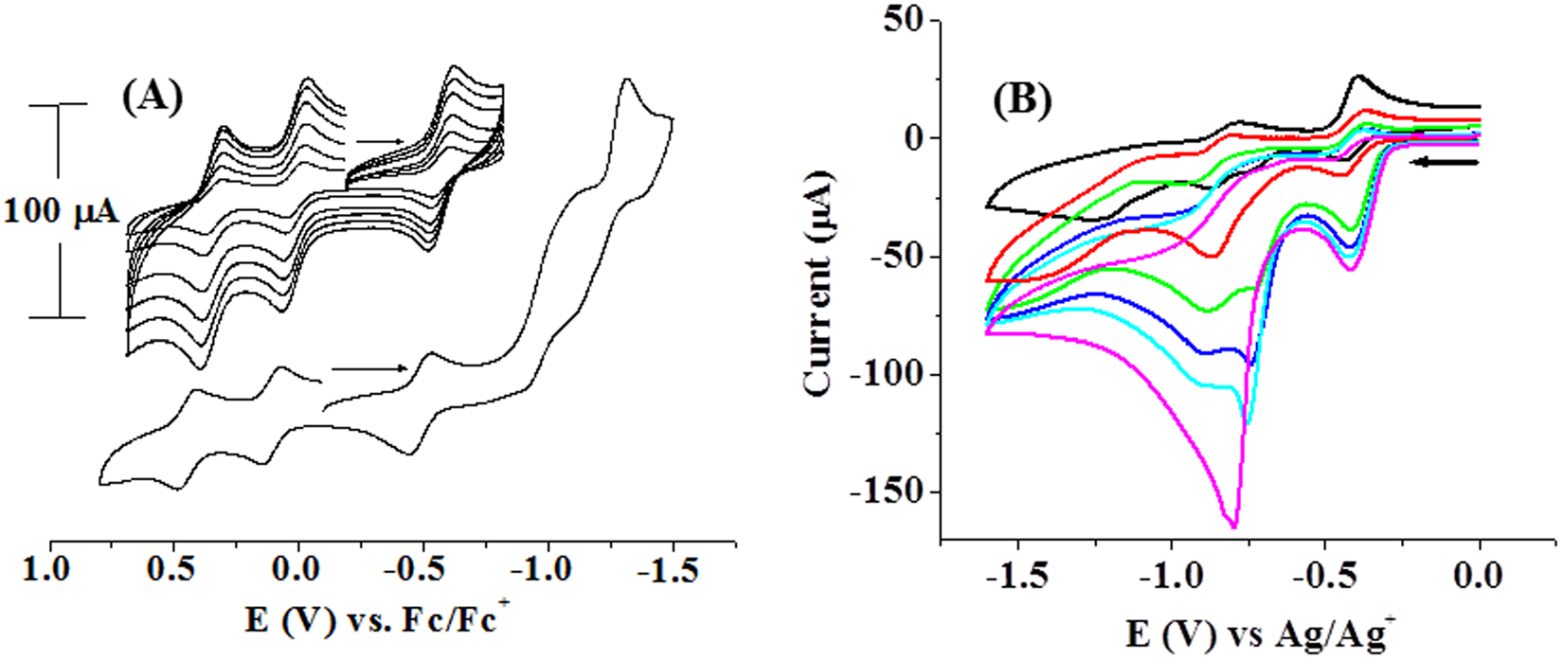
(**A**) Cyclic voltammogram of (1) (1 mM) in CH_2_Cl_2_ (Supporting electrolyte, NBu_4_ClO_4_ (0.2 M), GCE working, Pt wire auxillary and Ag/AgCl reference electrodes). Inset, reversible redox couples at different scan rates, 50–500 mV/sec. (**B**) Cyclic voltammograms of the cluster (1) (0.025 mM, black) in CH_3_CN as a function of increasing concentrations of added *p*-TsOH (0.25 M, 0.05 ml additions each) at a scan rate of 100 mVs^−1^. Supporting electrolyte, NBu_4_PF_6_ (0.2 M), GCE working, Pt wire auxillary and Ag/AgCl reference electrodes.

The Fe_4_S_4_ cubane type cluster (**1**) undergoes three reversible one electron redox processes around E_1/2_, +0.45 V, +0.11 V and −0.49 V (ΔE, 60 mV) and two quasi-reversible redox processes around E, −1.01 V and −1.22 V in CH_2_Cl_2_ (Fig. 6). The waves at E, −0.49 V, −1.01 V and −1.22 V can be assigned to the DMET ligand based redox processes since these were observed also in other related iron(III) dithiolene complexes and a nickel(II) complex of the DMET ligand^30–32^. The reversible redox process occurring near zero (E_1/2_, +0.107 V) can be assigned to Fe^3+^/Fe^2+^ and the one occurring at E_1/2_, +0.45 V can be assigned to Fe^2+^/Fe^3+^ redox process. These assignments of the redox waves of the cluster (**1**) have been done based on comparison of cyclic voltammetric profiles of various Fe_4_S_4_ clusters, iron(II)/iron(III) and nickel(II)complexes of similar dithiolene ligands including classical tetrahedral Fe_4_S_4_ clusters of Holm *et al*.^50–59^.

The complex (**1**) electro-catalyzes hydrogen evolution from *p*-toluene sulfonic acid (TsOH) in CH_3_CN. On addition of TSOH (0.25 M, 0.05 ml) to the cluster (**1**) in CH_3_CN, the negative current at potential E, −0.72 V was increasing as shown in Fig. 6B. But the reversible redox couples around E_p_, +0.445 V and Ep, +0.107 V were unaffected by the addition of TsOH. This indicates that the preferential sites of protonation could be the sulfur donors of the radical containing monoanionic dithiolate. The increase in current is due to the reduction of TsOH protons followed by the evolution of hydrogen gas. Controlled potential electrolysis of a mixture of the cluster (**1**) (0.025 mmol) and TsOH (0.25 mmol) was carried out in CH_3_CN at E_p_, −0.8 V. A net charge of 29 mC passed over a period of 2 minutes. Head space analysis of the electrochemical cell by gas chromatography confirmed the presence of H_2_ gas. The cluster (**1**, 0.025 mmol) consumed 0.25 mmol of TsOH and the TON (turnover number) of (**1**) in CH_3_CN is 400. The H_2_ evolution occurring at E_p_, −0.8 V using TsOH is proposed to be promoted by a S-radical based process. The iron bound monoanionic dithiolate type S-donor sites of DMET can be protonated upon addition of TSOH and reduce protons to H_2_ on application of electric potential. In the process, the monoanionic dithiolate S- donors can in turn get oxidized to a fully oxidized, neutral di-radical ligand. The reduction of protons coupled to the oxidation of monoanionic dithiolate to neutral di-radical ligand is modulated by the iron center. Because similar DMET complexes of several other transition metal ions do not catalyze proton reduction at such a low reduction potential, *viz*., E_p_, 0.72 V.

**Figure 7:**
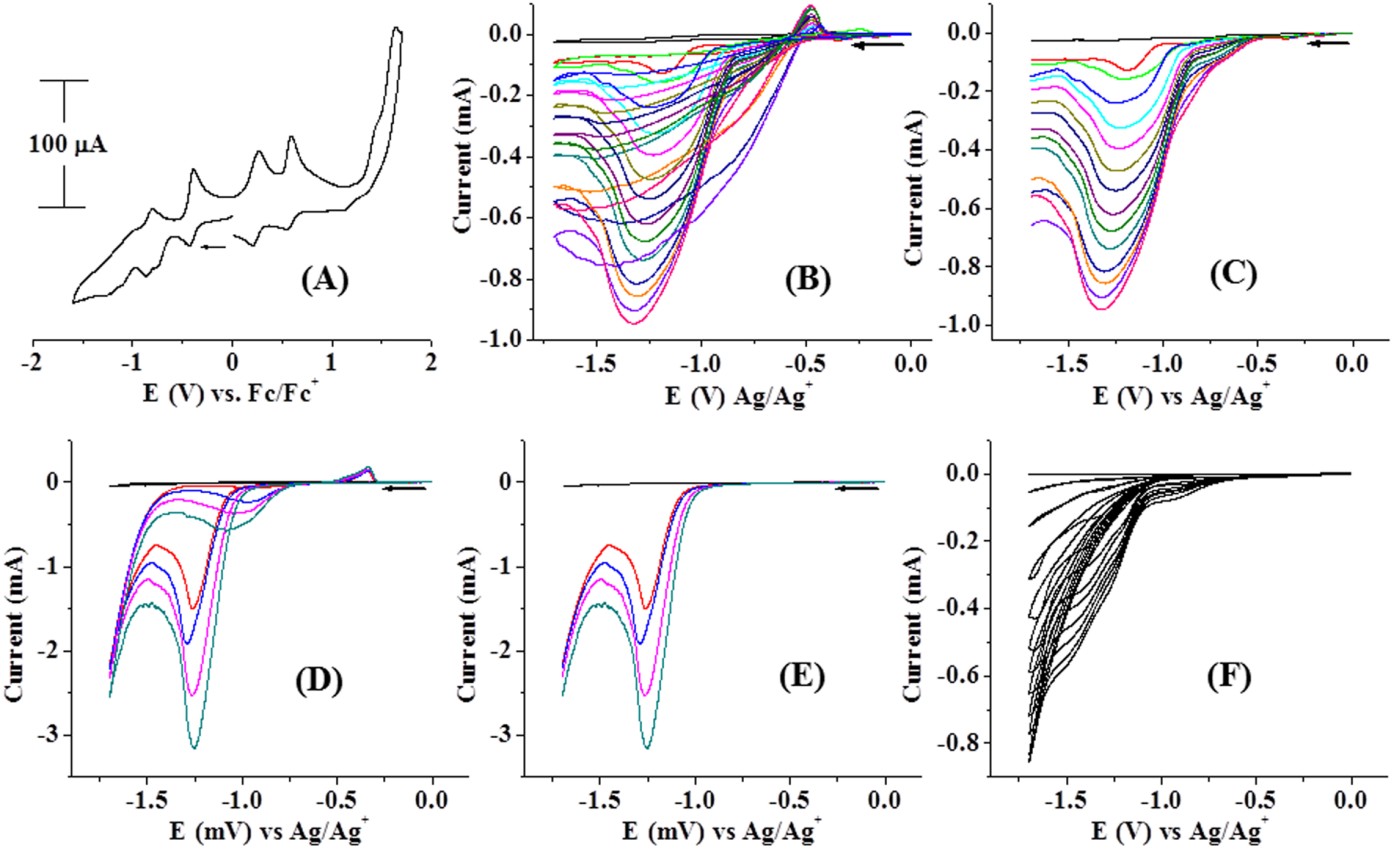
(**A**) Cyclic voltammogram of the graphene composite (**1**@graphene) (1 mM) in CH_3_CN scan rate of 100 mVs^−1^ (Supporting electrolyte, NBu_4_PF_6_/CH_3_CN or KNO_3_/H_2_O (0.2 M), GCE working, Pt wire auxillary and Ag/AgCl reference electrodes). (**B**) Cyclic voltammograms as a function of increasing concentrations of added *p*-TsOH in CH_3_CN. (0.5 M *p*-TsOH, 0.1 mL each in CH_3_CN), (**C**) Displaying only forward reduction waves for clarity, (**D**) 0.5 M *p*-TsOH, 0.1 mL each in water, (**E**) Displaying only forward reduction waves for clarity, (**F**) Addition of 0.5 M *p*-TsOH, 0.1 mL each in CH_3_CN to functionalized graphene only.

The cyclic voltammogram of the composite, **1**@graphene is similar to that of the cluster (**1**) as shown in Fig. 7A. It displays three reversible one electron redox processes at E_p_, −0.41 V, +0.23 V and +0.57 V in CH_3_CN which are assigned to S-radical based Fe^2+^/Fe^3+^ redox processes as described above for the pure cluster in CH_2_Cl_2_ (*vide supra*). The composite, **1**@graphene electrocatalyzes TsOH-proton reduction to H_2_ at E_p_, −1.19 V in CH_3_CN as a solvent. On addition of 0.4g of TsOH (2 mmol in 2 mL CH_3_CN), overall current of −0.95 mA passed at E, −1.19 V. The increase in current as a function of added TsOH solutions in CH_3_CN (1 M, 0.1 ml each) are shown in Fig. 7B and for clarity only the forward scans are displayed in Fig. 7C. The composite electrocatalyzes the above mentioned reduction to H_2_ at the same potential, E_p_, −1.2 V in water as a solvent with better catalytic efficiency and higher current output. On addition of 0.1 g of TsOH (0.5 mmol in 1 mL H_2_O), overall current of −3.15 mA passed at E, −1.2 V as shown in Fig. 7D and for clarity only the forward scans are displayed in Fig. 7E. The composite (0.05 g, 0.025 mmol with respect to Fe_4_S_4_ cubane cluster, **1**) consumed 2 mmol of TsOH with a turnover number of 3200. Formation of bubbles and brisk effervescence were observed upon addition CH_3_CN (1mL) to the electrochemical cell containing the composite **1**@graphene and TsOH in water. This was confirmed to be H_2_ gas by head space analysis by gas chromatography^30^. The stability of the Fe_4_S_4_ cubane type cluster and its composite, **1**@graphene are confirmed in de-aerated water under argon due to the insolubility of (**1**) in water. The cluster (**1**) was extracted from the composite after cyclic voltammetric experiments by ultrasonication in CH_3_CN followed by centrifugation and evapouration to dryness. The elemental analysis of the residue indicated the presence of carbon, hydrogen, nitrogen and sulphur according to the percentage elemental composition of the cluster (**1**). The composite, **1**@graphene isolated from water medium displayed an ESI-MS (−ve) signal at m/z, 1514. The IR spectrum of **1**@graphene after catalysis is dominated by peaks due to ν_NO3_ (KNO_3_, supporting electrolyte) and ν_S=O_ (tosic acid) stretching at 1371 cm^−1^, 1177 cm^−1^ and 1120 cm^−1^. But weak signals at 1720 & 1644 cm^−1^ (-COO of dithiolene) and at 1528 & 1490 cm^−1^ (-C=C- of dithiolene) coupled with the elemental analytical data indicate that the Fe_4_S_4_ cluster is intact. The cluster@graphene is confirmed to be stable in the solid state in water where as it is prone for attack only when it is dissolved in CH_3_CN or even in a mixture of H_2_O-CH_3_CN. This is due to the fact that in CH_3_CN, the composite functions as an emulsion of graphene in the CH_3_CN solution of (**1**) where as in water, it is an emulsion in graphene solution. The composite remains intact with indefinite durability and it is well behaved in pure water. But in a mixture of H_2_O-CH_3_CN, both the cluster (**1**) and graphene get into solution. The composite, **1**@graphene shows an enhanced catalytic activity (TON, 3200) as compared to the pure Fe_4_S_4_ cluster (**1**) alone (TON, 400). This could be due to *in situ* reduction of the oxidized Fe_4_S_4_ cluster by graphene. As much as the cluster (**1**) gets oxidized after proton reduction, that much can be reduced on the graphene matrix immediately. The graphene matrix can function similar to an external sacrificial electron donor^60^. Only graphene in the absence of the Fe_4_S_4_ cubane type cluster electrocatalyzes proton reduction at a higher negative potential, *ca*., −1.7 V as shown in Fig. 7F. But the catalysis onsets at the reduction potential, *ca*. Ep, −0.41 V itself. Under similar experimental conditions, only TsOH, did not show any response on a GCE in the potential range, E, 0.0 V to −1.8 V (supporting information).

In summary, an ideal catalytic material for the reduction of TsOH protons to H_2_ gas in water has been achieved by immobilizing the Fe_4_S_4_ cubane type cluster, [PPh_4_]_2_[Fe_4_S_4_(DMET)_4_] (**1**) on a functionalized graphene support. A high current output (3200 TON) and an extreme stability of the catalytic material in water was concluded from the cyclic voltammetric and ESI-MS experiments. Only Fe_4_S_4_ cluster, (**1**) electrocatalyzes the same reduction reaction with low catalytic efficiency (400 TON) and concomitant decomposition to dimeric compound, [PPh_4_]_2_[Fe^III^(DMET)_2_]_2_. The Fe_4_S_4_ cubane type cluster was synthesized in a novel synthetic route and structurally characterized. The cluster (**1**) is EPR silent and it displays reversible, one electron redox waves around E_1/2_, +0.107 V and at +0.45 V) which could be assigned to the Fe^3+^/Fe^2+^ and Fe^2+^/Fe^3+^ redox processes.

## Methods

### Synthesis of [PPh_4_]_2_[Fe_4_S_4_{S_2_C_2_(COOCH_3_)_2_}_4_] (1)

The reaction was done under Schlenk conditions. Acetonitrile (40 ml) was purged with argon gas for 30 min. and iron(II)-polysulfide, [PPh_4_]_4_[Fe^II^_2_S_12_] (Electronic Supplementary information) (0.500 g) was added into it. The suspension was stirred for 20 min. followed by the addition of dimethyl acetylene carboxylate (0.5 ml) and freshly purchased lithium sulfide (Li_2_S, 0.15 g) under argon. The black colored reaction mixture was stirred for 8h at room temperature and then diethylether (100 ml) was added. The reaction flask was closed tightly and allowed to stand at 10 °C for two days under argon. Dark green crystals were formed which were filtered and stored under argon. Dark green single crystals suitable for X-ray diffraction were obtained by layering of diethylether onto acetonitrile solution of the complex under argon. Data for (**1**): Yield, 0.6 g, C, H, N elemental analysis calculated for [PPh_4_]_2_[Fe_4_S_4_{S_2_C_2_(COOCH_3_)_2_}_4_].2H_2_O, C, 45.72, H, 3.62; Found, C, 45.53, H, 3.82. ESI-MS (negative) in CH_3_CN; m/z, 1514 {[PPh_4_][Fe_4_^III^(μ_3_–S)_4_(S_2_C_2_(COOCH_3_)_2_)_4_]}^−^; ESI-MS (positive), m/z, 339 [PPh_4_]^+^. FT-IR (KBr disc, cm^−1^): 3400(br), 2945(m), 1714 (s), 1693 (s), 1433(s), 1238 (s), 1212 (m), 1084 (m), 995(m), 722 (s), 688 (s), 526(s) (br, broad, w, weak, m, medium, s, strong). UV-visible in CH_2_Cl_2_ [λ/nm (ε/M^−1^cm^−1^)]: 823 (2200), 582 (4500), 467 (5600). EPR silent, μ_eff_ = 0.8 μ_B_ at 298K. E ½ = +0.445 V (ΔE = 60 mV), +0.107 V (ΔE = 60 mV) and −0.494 V (ΔE = 60 mV) vs. Ag/AgCl; E, (quasi-reversible), −1.005 V and −1.219 V vs. Ag/AgCl in CH_2_Cl_2_-0.2M NBu_4_ClO_4_ with GC working electrode.

#### Preparation of functionalized grapheme

Graphite powder (0.5 g) was taken in THF (80 mL) and water (20 mL), stirred at 38 C for 2 h and ultra-sonicated for 10 h. The solvents were decanted after centrifugation and the residue was dried thoroughly. This residue was treated carefully dropwise with concentrated H_2_SO_4_ (30 mL) and fuming nitric acid (10 mL) at 0 °C. The reaction mixture was heated under reflux for 12 h and allowed to stand at 38 °C for 10 h. The supernatant acid layer was decanted and the residue was washed thoroughly with water by centrifugation and dried. FT-IR (ν, cm^−1^): 1718 (br.), 1600 (w).

#### Preparation of the Composite, 1@graphene

The Fe_4_S_4_ cubane type cluster (**1**, 0.05 g) in degassed LC-MS grade CH_3_CN (12 mL) was mixed with functionalized graphene (0.05 g) in degassed LC-MS grade H_2_O (8 mL) and ultra-sonicated in closed sample vial under argon atmosphere for 3 h. The reaction mixture was evaporated to dryness and the residue was used in catalytic experiments. FT-IR (ν, cm^−1^): 3324(br.), 1616 (br.), 1610 (w), 1433(w), 1364 (w), 1233 (w), 1103 (m), 997 (m) 751 (w), 593 (w).

### X-ray crystallographic measurement of (1)

The dark green single crystals of the complex (**1**) were obtained by layering of diethylether onto acetonitrile solution of the complex under argon and isolated as [PPh_4_]_2_[Fe_4_^III^(μ_3_–S)_4_(DMET)_2_^4−^((DMET)^1−∙^)_2_]^2−^. (CH_3_CH_2_)_2_O. 2H_2_O. The intensity data for single crystals of (**1**) was collected at 120 K on a Bruker AXS Smart APEX CCD diffractometer with graphite monochromated MoK_α_ radiation (0.71073 Ǻ). Data reduction and absorption corrections were done using SAINTPLUS program package. The structure was solved by direct and conventional Fourier methods and refined on F^2^ by full-matrix least-squares technique using SHELXTL program package^61^. All non-hydrogen atoms were refined anisotropically, H atoms at idealized positions in riding mode. structure data for (**1)**: C_76_H_64_Fe_4_O_19_P_2_S_12_, Mr = 1951.33, Monoclinic, P2/c, a = 13.541(5), b = 13.376(5), c = 24.923(5) (Ǻ), β = 98.859(5)°, Volume = 4460(3) Ǻ3, Z = 4, ρ_calcd_ = 1.453 Mg/m3, μ(MoKα) = 1.018 mm^−1^, F(000) = 1996, Unique reflections, 22403 at 120 K, Observed reflections, 7840, Parameters, 503, GOF = 1.034, R1 = 0.0632, wR2 = 0.1796. CCDC-871183 of the complex (**1)** can be downloaded from Cambridge crystallographic data center.

## Electronic supplementary material

Supplementary Information
