# Supplementary material for: Fe4S4 Cubane Type Cluster Immobilized on a Graphene Support: A High Performance H2 Evolution Catalysis in Acidic Water
Source: Sci Rep. 2017 Dec 5;7:16948. doi: 10.1038/s41598-017-17121-7 (PMC5717245; doi:10.1038/s41598-017-17121-7)
Supplement: Supplementary file 1 — Supplementary Information [file 41598_2017_17121_MOESM1_ESM.pdf]

Electronic supplementary information

Fe_4S_4 Cubane Type Cluster Immobilized on a Graphene Support: A High Performance H_2 Evolution Catalysis in Acidic Water

Ameerunisha Begum,[1,*] Aasif Hassan Sheikh[1], Golam Moula[2] and Sabyasachi Sarkar[2]

[1]Department of Chemistry, Faculty of Science, Jamia Hamdard University, New Delhi – 110062, India; [2]Department of Chemistry, Indian Institute for Engineering Science and Technology, Shibpur, Howrah – 710008, West Bengal, India.

(I) Materials and methods

All solvents and chemicals were purchased from commercial sources. Dimethyl acetylene carboxylate, anhydrous ferric chloride, lithium sulfide, LC-MS grade acetonitrile, water and tetraphenyl phosphonium bromide were purchased from Aldrich (USA). The elemental analysis was performed on Perkin-Elmer 2400 microanalyzer. Electrospray mass spectrometric (ESI-negative and ESI-positive) measurement was done on Waters micromass QTOF Premier mass spectrometer. The electronic absorption spectroscopic measurements were done on a Perkin-Elmer Lambda 35 spectrometer. Infrared spectra were measured on a Bruker Vertex 70 FT-IR spectrometer. X-band EPR spectral measurements were done using a Bruker EMX Spectrometer. Vibrating sample magnetic moment measurement was conducted on EV-7 VSM ADE-DMS-Magnetic instrument within the field range of -17500 to +17500 Oe at room temperature (294 - 300 K). The data were corrected for diamagnetism of the sample holder and diamagnetic corrections for the complex (**1**) were deduced from Pascal's constants. Cyclic voltammetric studies were carried out at 298 K on a BASi Epsilon-Eclipse Bioanalytical systems in CH_3CN or H_2O or CH_2Cl_2. Glassy carbon working, platinum wire auxiliary and Ag/AgCl with 3.0 M KCl reference electrodes were used. Tetrabutylammonium perchlorate (TBAP) or Tetrabutyl ammonium hexafluoro phosphate (TBA-PF_6) or potassium nitrate of 0.2 M concentration were used as supporting electrolytes in CH_2Cl_2, CH_3CN and H_2O respectively. Gas chromatographic analysis of H_2 gas was performed on Agilent Technologies 7890A GC system using N_2 carrier gas. A mixture of the complex **1**@graphene (~0.025 mmol) and TsOH (0.5 mole) in CH_3CN (NBu_4PF_6 supporting electrolyte, 0.2M) was purged with nitrogen for 15 min. A 2 ml syringe was inserted into the compartment of electrochemical cell and the argon gas supply inlet was closed. The reaction mixture was subjected to controlled potential electrolysis at -1.2 V, the gas

bubbles formed at the GC working electrode surface were tapped off to the surface and the gas over the solution was taken into the syringe which was then injected into the GC system. In a separate experiment, the composite **1**@graphene (0.05 g; ~0.025 mmol) and TsOH (0.6 g; 3 mmoles) were taken in degassed water (12 mL) in the presence of KNO_3 (0.2 moles) and left for 30 min. To this CH_3CN (1 mL) was added and a brisk effervescence and gas bubbles were observed. The gas was collected in syringe and analyzed by gas chromatography.

(II) Experimental section

Preparation of methanolic solution of potassium polysulfide:

H_2S gas was passed into a mixture of elemental sulphur (20 g) and KOH (4 g) in methanol (100 ml) for a period of 2h with continuous stirring. The reaction solution changed from light yellow to deep red. This red colored polysulfide solution was filtered and used immeditately for further reactions.

Preparation of iron(II)-polysulfide $[PPh_4]_4[Fe^{II}_2S_{12}]$:

The red colored methanolic polysulfide solution (50 ml) described above was treated with tetraphenyl phosponium bromide (4 g) and then anhydrous $FeCl_3$ (1 g) was added portion wise. The reaction mixture was stirred for at room temperature for 3 h and then heated on a steam bath for 2h. The black prepcipitate thus formed was filtered and washed thoroughly with 100 ml each of water, methanol, toluene and diethyl ether. Then it was dried thoroughly under vacuum. Closed tightly and stored at -4° C in a refrigerator. Yield, 3.8 g, FT-IR (KBr disc, cm^{-1}): 3053(w), 1584 (w), 1435(s), 1106(s), 721(s), 687(s), 525(s) (w, weak, s, strong).

(III) IR spectrum of the complex (1) in KBr phase:

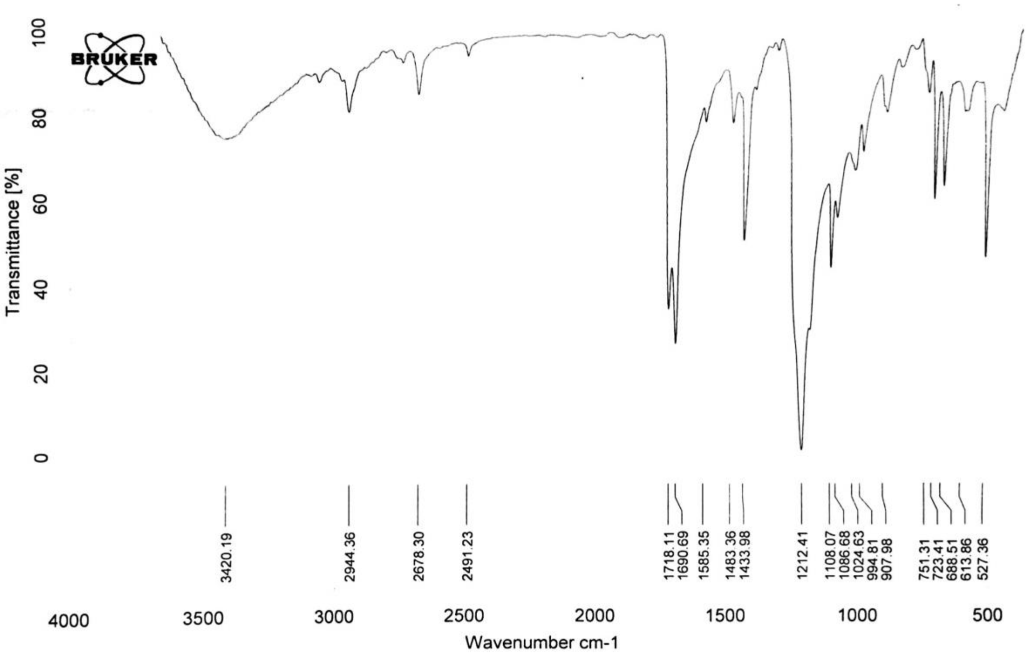

Figure 1. IR spectrum of the Fe_4S_4 cluster (**1**).

(IV) IR spectrum of functionalized graphene:

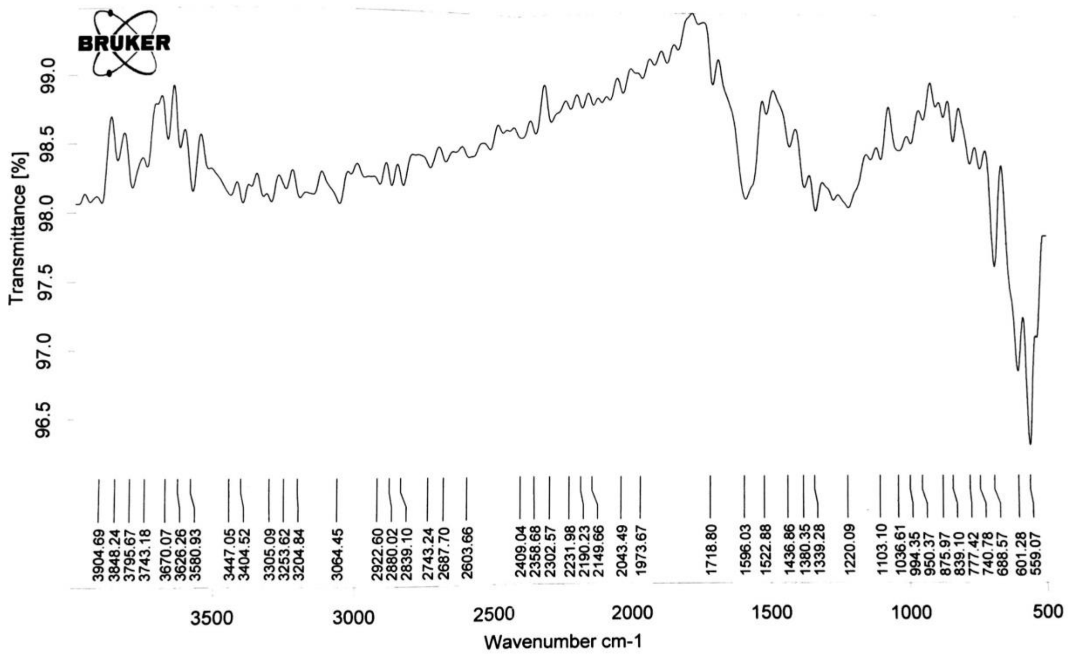

Figure 2. IR spectrum of functionalized graphene.

(V) IR spectrum of 1@graphene:

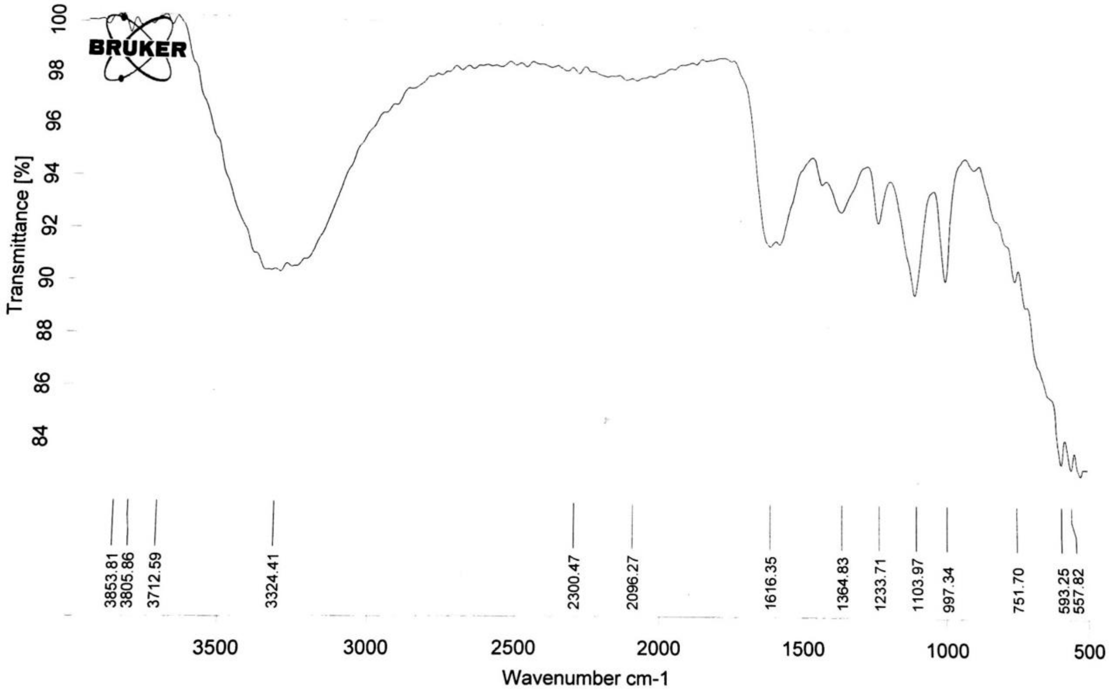

Figure 3. IR spectrum of **1**@graphene.

(VI) IR spectrum of 1@graphene after catalysis:

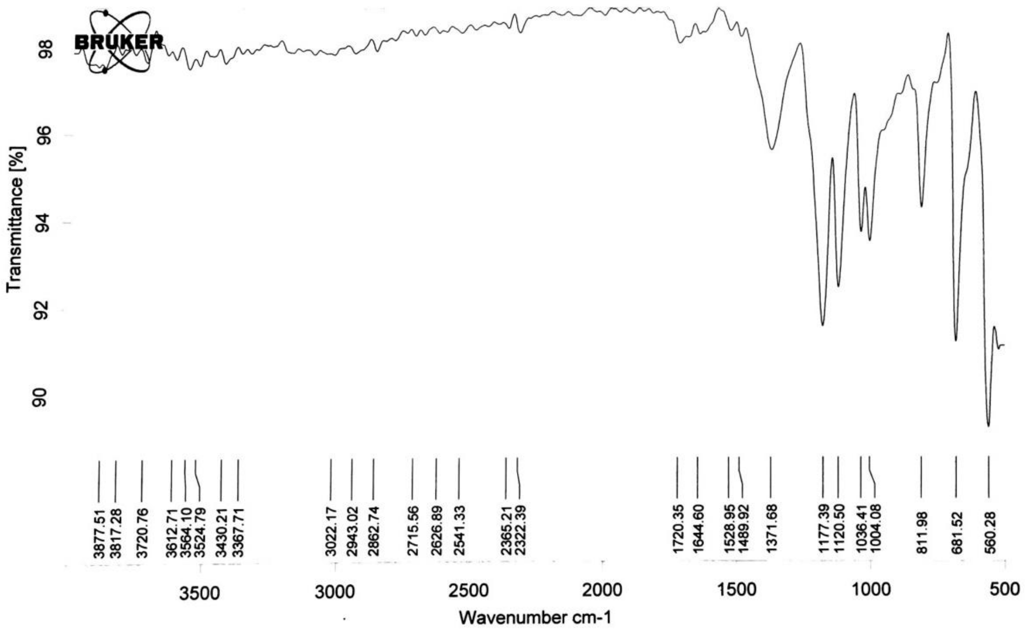

Figure 4. IR spectrum of **1**@graphene after catalysis.

(VII) ESI-MS (positive) of the complex (1) in CH_3CN showing the existence of $[PPh_4]^+$ ions in CH_3CN:

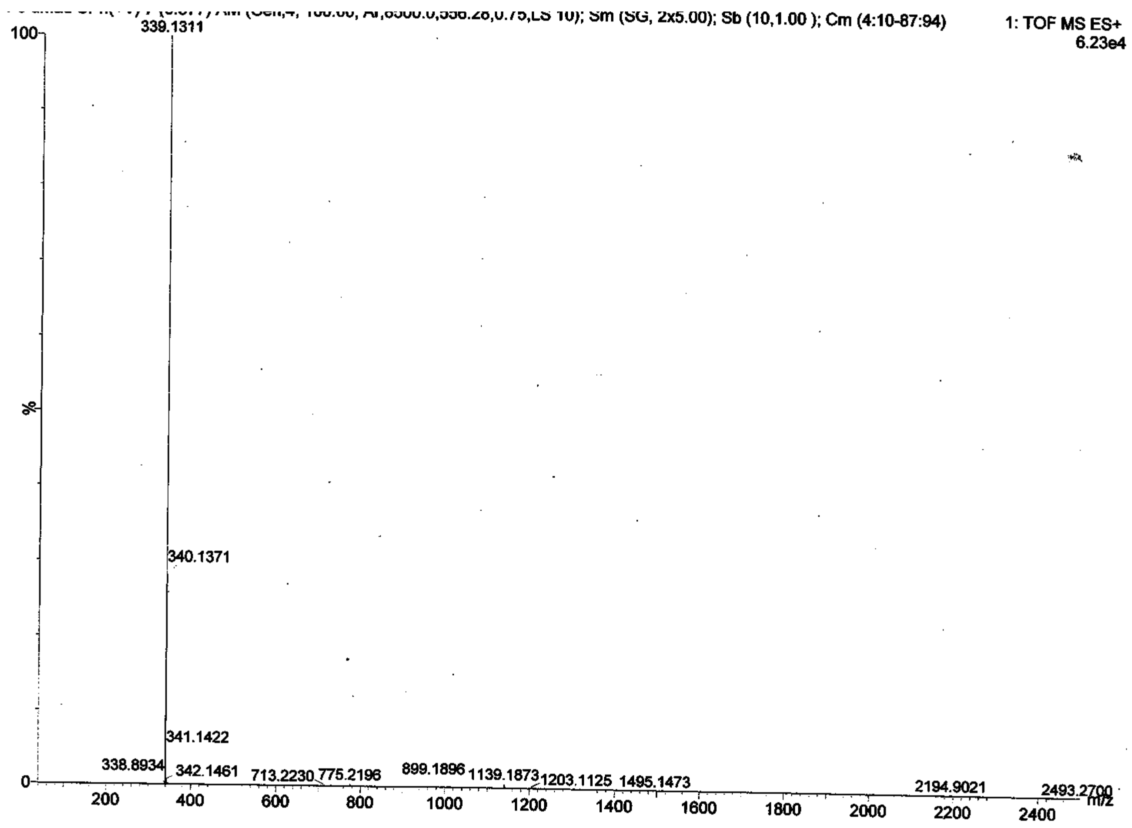

Figure 2. ESI-MS (positive) of the complex (**1**) in CH_3CN.

(VIII) Space filling model of structure of (1) and (2).

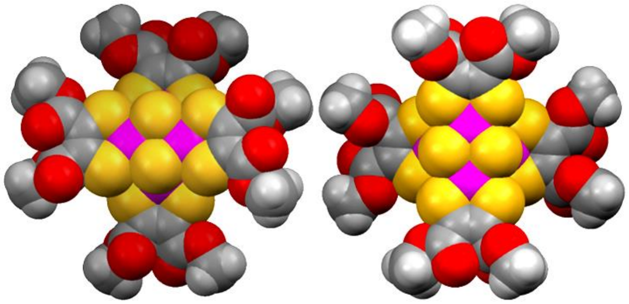

(a) (b)

Figure 4. The structure of the anionic part of the complex (**1**) in space filling model. (a) front view; (b) rear view. Note the identical geometrical environment around all of the four iron centers (magenta).

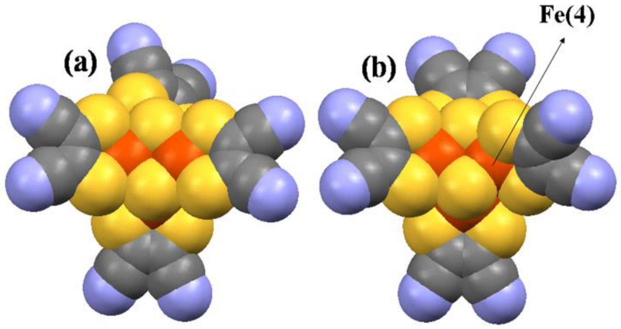

Figure 5. The structure of the anionic part of the tetra anionic cluster, (**2**) reported in ref. 32. a) front view and (b) rear view. Note the distorted iron center Fe(4).

(IX) Cyclic voltammograms of (1) in CH_2Cl_2 and in the presence of TsOH

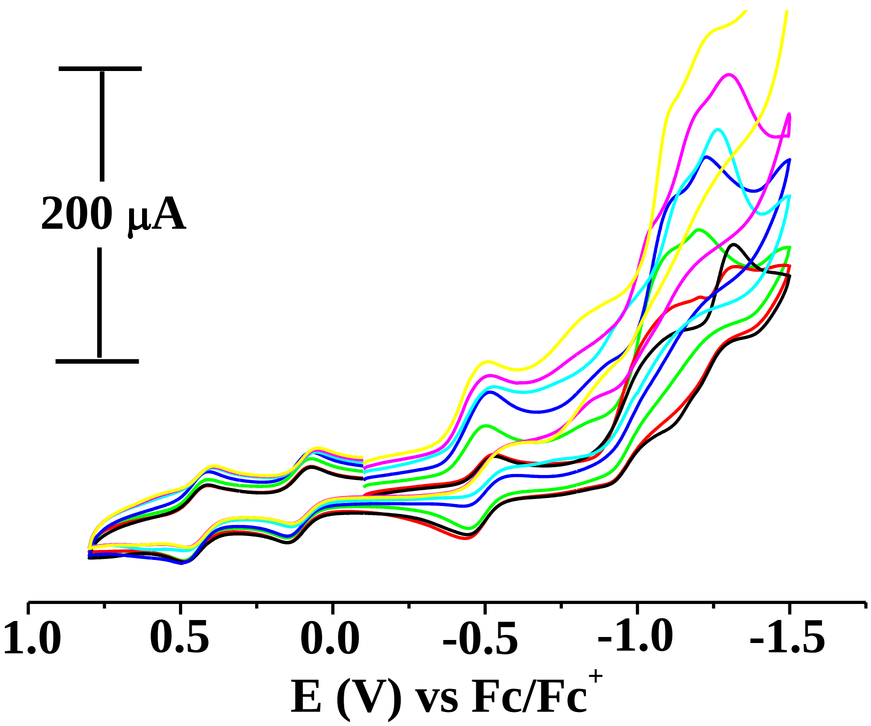

Figure 6. Cyclic voltammograms of the cluster (**1**) (1 mM, red) in CH_2Cl_2 (12 ml) as a function of increasing concentrations (0.008 g additions each) of added *p*-TsOH at a scan rate of 100 mVs^{-1}. Supporting electrolyte, NBu_4ClO_4 (0.2 M), GCE working, Pt wire auxillary and Ag/AgCl reference electrodes.

(X) Cyclic voltammograms of (1) in DMF and in the presence of TsOH

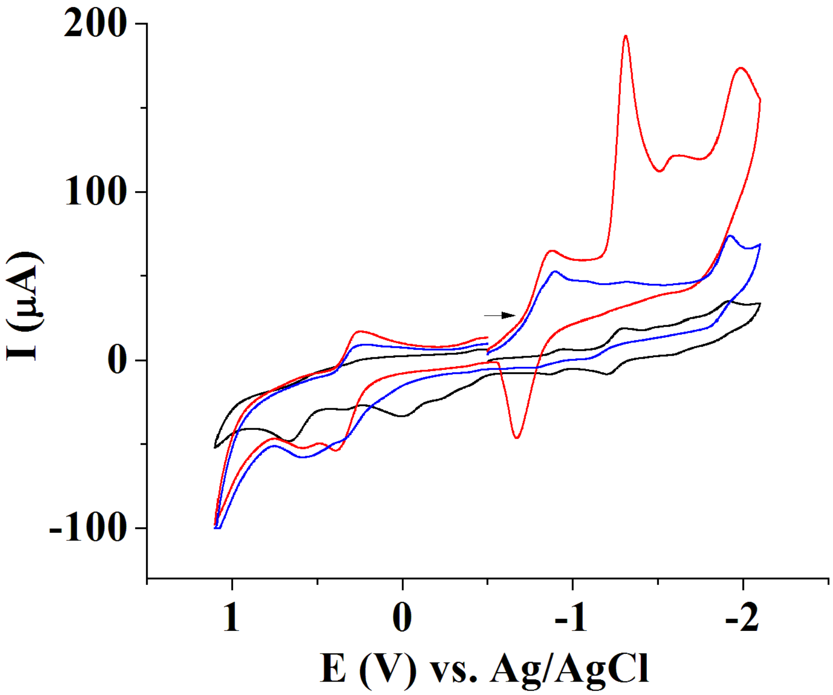

Figure 7. Cyclic voltammograms of the cluster (**1**) (1 mM, black) in DMF (12 ml) as a function of increasing concentration of TsOH at a scan rate of 100 mVs^{-1}. (blue, 0.095 g, 0.041 M; red, 0.19 g, 0.083 M). Supporting electrolyte, NBu_4ClO_4 (0.2 M), GCE working, Pt wire auxillary and Ag/AgCl reference electrodes.

(XI) Cyclic voltammograms of TsOH in CH_3CN as a function of increasing concentrations.

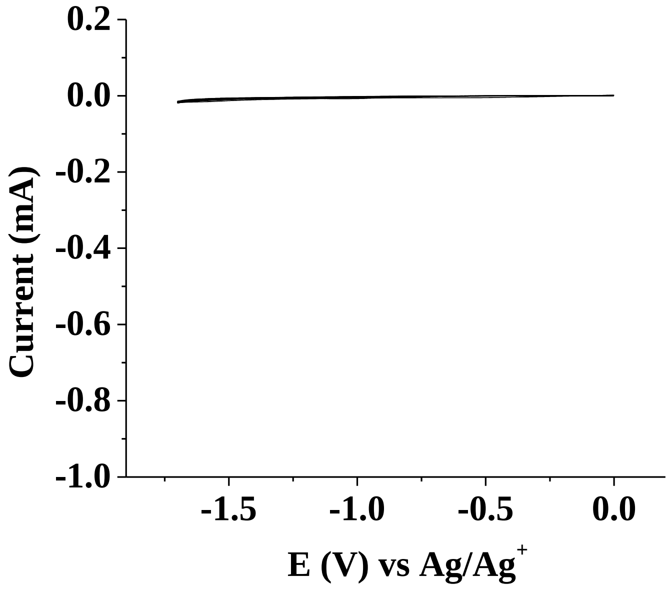

Figure 8. Cyclic voltammograms of TSOH in CH_3CN (12 ml) as a function of increasing concentrations at a scan rate of 100 mVs^{-1}. (black, 0.041 M; blue, 0.083 M; red, 0.125 M; green, 0.208 M). Supporting electrolyte, NBu_4ClO_4 (0.2 M), GCE working, Pt wire auxillary and Ag/AgCl reference electrodes.

(XII) Lattice packing in crystals of (1)

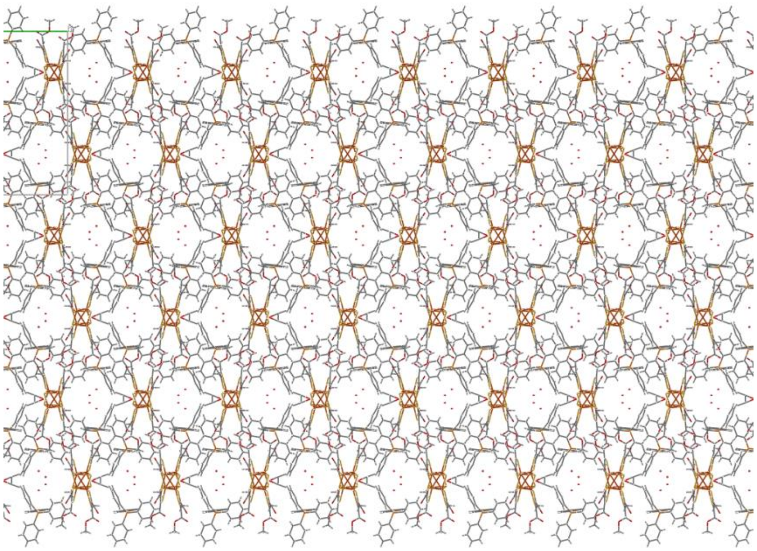

Figure 9. View of lattice packing in complex (**1**) along a-axis.

(XIII) Table 1. Crystal data for (1)

Compound	**(1)**			
Empirical formula	$C_{76}H_{64}Fe_4O_{19}P_2S_{12}$			
Formula weight	1951.33			
Temperature (K)	120			
Wave length MoKα (Å)	0.71073			
Crystal system	Monoclinic			
Space group	P2/c			
	a/(Å)	13.541(5)	α/(º)	90.00
	b/(Å)	13.376(5)	β/(º)	98.859(5)
	c/(Å)	24.923(5)	γ/(º)	90.00
Volume ($Å^3$)/Z	4460(3)/4			
Density (Mg/m^3)	1.453			
Absorption coefficient (mm^{-1})	1.018			
F(000)	1996			

θ range for data collection (º)	2.07 to 25.00
Unique reflections	22403
Observed reflections	7840
Absorption correction	Empirical
Parameters/restraints	503/14
Goodness of fit on F^2	1.034
Final R_1 values [I>2σ(I)]	0.0632
Final $wR(F^2)$ values [I>2σ(I)]	0.1796
CCDC number	871183

(XIV) Table 2. Selected bond distances (Å) in the structure of the complex (1)

Fe(1)-S(1)	2.1827(18)	Fe(1)-S(2)	2.2012(16)
Fe(1)-S(3)	2.1567(18)	Fe(1)-S(4)	2.2443(17)
Fe(1)-Fe(2)	2.6952(13)	Fe(1)-Fe(2)	2.7226(12)
Fe(2)-S(4)	2.1574(18)	Fe(2)-S(6)	2.1911(16)
Fe(2)-S(5)	2.2018(17)	Fe(2)-S(3)	2.2401(16)
S(1)-C(1)	1.734(6)	S(2)-C(2)	1.718(6)
S(5)-C(7)	1.737(6)	S(6)-C(8)	1.721(6)

CIF of (1)

```
data_6jana

_audit_creation_method            SHELXL-97
_chemical_name_systematic
;
 ?
;
_chemical_name_common             ?
_chemical_melting_point           ?
_chemical_formula_moiety          'C76 H64 Fe4 O19 P2 S12'
_chemical_formula_sum
 'C76 H64 Fe4 O19 P2 S12'
_chemical_formula_weight          1951.33

loop_
 _atom_type_symbol
 _atom_type_description
 _atom_type_scat_dispersion_real
 _atom_type_scat_dispersion_imag
 _atom_type_scat_source
 'C'  'C'   0.0033   0.0016
 'International Tables Vol C Tables 4.2.6.8 and 6.1.1.4'
 'H'  'H'   0.0000   0.0000
 'International Tables Vol C Tables 4.2.6.8 and 6.1.1.4'
 'O'  'O'   0.0106   0.0060
 'International Tables Vol C Tables 4.2.6.8 and 6.1.1.4'
 'P'  'P'   0.1023   0.0942
 'International Tables Vol C Tables 4.2.6.8 and 6.1.1.4'
 'S'  'S'   0.1246   0.1234
 'International Tables Vol C Tables 4.2.6.8 and 6.1.1.4'
 'Fe'  'Fe'   0.3463   0.8444
 'International Tables Vol C Tables 4.2.6.8 and 6.1.1.4'

_symmetry_cell_setting            monoclinic
_symmetry_space_group_name_H-M    P2/c

loop_
 _symmetry_equiv_pos_as_xyz
 'x, y, z'
 'x, -y, z+1/2'
 '-x, -y, -z'
 '-x, y, -z-1/2'

_cell_length_a                    13.541(5)
_cell_length_b                    13.376(5)
_cell_length_c                    24.923(5)
_cell_angle_alpha                 90.000(5)
_cell_angle_beta                  98.859(5)
_cell_angle_gamma                 90.000(5)
_cell_volume                      4460(3)
_cell_formula_units_Z             2
```

```
_cell_measurement_temperature     100(2)
_cell_measurement_reflns_used     3991
_cell_measurement_theta_min       2.25
_cell_measurement_theta_max       28.27

_exptl_crystal_description        block
_exptl_crystal_colour             black
_exptl_crystal_size_max           0.06
_exptl_crystal_size_mid           0.05
_exptl_crystal_size_min           0.04
_exptl_crystal_density_meas       ?
_exptl_crystal_density_diffrn     1.453
_exptl_crystal_density_method     'not measured'
_exptl_crystal_F_000              1996
_exptl_absorpt_coefficient_mu     1.018
_exptl_absorpt_correction_type    'empirical'
_exptl_absorpt_correction_T_min   0.941
_exptl_absorpt_correction_T_max   0.960
_exptl_absorpt_process_details    'SADABS (Bruker, 2002)'

_exptl_special_details
;
 ?
;

_diffrn_ambient_temperature       100(2)
_diffrn_radiation_wavelength      0.71069
_diffrn_radiation_type            MoK\a
_diffrn_radiation_source          'fine-focus sealed tube'
_diffrn_radiation_monochromator   graphite
_diffrn_measurement_device_type   'CCD area detector'
_diffrn_measurement_method        'Omega scans'
_diffrn_detector_area_resol_mean  ?
_diffrn_standards_number          ?
_diffrn_standards_interval_count  ?
_diffrn_standards_interval_time   ?
_diffrn_standards_decay_%         ?
_diffrn_reflns_number             22403
_diffrn_reflns_av_R_equivalents   0.0604
_diffrn_reflns_av_sigmaI/netI     0.0733
_diffrn_reflns_limit_h_min        -16
_diffrn_reflns_limit_h_max        15
_diffrn_reflns_limit_k_min        -14
_diffrn_reflns_limit_k_max        15
_diffrn_reflns_limit_l_min        -29
_diffrn_reflns_limit_l_max        23
_diffrn_reflns_theta_min          2.07
_diffrn_reflns_theta_max          25.00
_reflns_number_total              7840
_reflns_number_gt                 5540
_reflns_threshold_expression      >2sigma(I)

_computing_data_collection        'Bruker SMART'
_computing_cell_refinement        'Bruker SMART'
```

```
_computing_data_reduction         'Bruker SAINT'
_computing_structure_solution     'Bruker SHELXTL'
_computing_structure_refinement   'SHELXL-97 (Sheldrick, 1997)'
_computing_molecular_graphics     ORTEP-3'
_computing_publication_material   'SHELXL-97 (Sheldrick, 1997)'

_refine_special_details
;
 Refinement of F^2^ against ALL reflections.  The weighted R-factor wR and
 goodness of fit S are based on F^2^, conventional R-factors R are based
 on F, with F set to zero for negative F^2^. The threshold expression of
 F^2^ > 2sigma(F^2^) is used only for calculating R-factors(gt) etc. and
is
 not relevant to the choice of reflections for refinement.  R-factors
based
 on F^2^ are statistically about twice as large as those based on F, and
R-
 factors based on ALL data will be even larger.
;

_refine_ls_structure_factor_coef  Fsqd
_refine_ls_matrix_type            full
_refine_ls_weighting_scheme       calc
_refine_ls_weighting_details
 'calc w=1/[\s^2^(Fo^2^)+(0.1230P)^2^+3.4055P] where P=(Fo^2^+2Fc^2^)/3'
_atom_sites_solution_primary      direct
_atom_sites_solution_secondary    difmap
_atom_sites_solution_hydrogens    geom
_refine_ls_hydrogen_treatment     constr
_refine_ls_extinction_method      none
_refine_ls_extinction_coef        ?
_refine_ls_number_reflns          7840
_refine_ls_number_parameters      503
_refine_ls_number_restraints      14
_refine_ls_R_factor_all           0.0902
_refine_ls_R_factor_gt            0.0632
_refine_ls_wR_factor_ref          0.2030
_refine_ls_wR_factor_gt           0.1796
_refine_ls_goodness_of_fit_ref    1.034
_refine_ls_restrained_S_all       1.061
_refine_ls_shift/su_max           0.006
_refine_ls_shift/su_mean          0.000

loop_
 _atom_site_label
 _atom_site_type_symbol
 _atom_site_fract_x
 _atom_site_fract_y
 _atom_site_fract_z
 _atom_site_U_iso_or_equiv
 _atom_site_adp_type
 _atom_site_occupancy
 _atom_site_symmetry_multiplicity
 _atom_site_calc_flag
```

```
 _atom_site_refinement_flags
 _atom_site_disorder_assembly
 _atom_site_disorder_group
Fe1 Fe 0.60484(6) 0.10432(6) 0.78989(3) 0.0155(2) Uani 1 1 d . . .
Fe2 Fe 0.44305(6) 0.21409(6) 0.80244(3) 0.0152(2) Uani 1 1 d . . .
O1 O 0.9720(3) -0.0226(3) 0.89048(17) 0.0253(10) Uani 1 1 d . . .
O2 O 0.9765(3) 0.0570(4) 0.81258(17) 0.0344(12) Uani 1 1 d . . .
O3 O 0.8759(3) 0.0999(3) 0.96735(16) 0.0226(9) Uani 1 1 d . . .
O4 O 0.8009(3) -0.0502(3) 0.96575(15) 0.0194(9) Uani 1 1 d . . .
O5 O 0.2121(3) 0.3453(3) 0.95057(16) 0.0272(10) Uani 1 1 d . . .
O6 O 0.1231(3) 0.2649(3) 0.88026(16) 0.0233(9) Uani 1 1 d . . .
O7 O 0.4559(3) 0.3529(3) 1.00023(15) 0.0223(9) Uani 1 1 d . . .
O8 O 0.3772(3) 0.2050(3) 1.00029(15) 0.0230(9) Uani 1 1 d . . .
S1 S 0.75755(11) 0.06823(12) 0.77871(6) 0.0215(4) Uani 1 1 d . . .
S2 S 0.64444(10) 0.06604(11) 0.87643(5) 0.0171(3) Uani 1 1 d . . .
S3 S 0.59334(10) 0.26488(11) 0.78400(5) 0.0165(3) Uani 1 1 d . . .
S4 S 0.44888(10) 0.05347(11) 0.79569(5) 0.0164(3) Uani 1 1 d . . .
S5 S 0.28924(11) 0.24629(11) 0.81579(6) 0.0180(3) Uani 1 1 d . . .
S6 S 0.49603(11) 0.25514(11) 0.88709(6) 0.0178(3) Uani 1 1 d . . .
P1 P 0.22594(11) 0.66765(11) 0.94698(6) 0.0177(3) Uani 1 1 d . . .
C1 C 0.8218(4) 0.0465(4) 0.8434(2) 0.0166(12) Uani 1 1 d . . .
C2 C 0.7712(4) 0.0460(4) 0.8867(2) 0.0158(12) Uani 1 1 d . . .
C3 C 0.9302(4) 0.0217(4) 0.8520(2) 0.0193(13) Uani 1 1 d . . .
C4 C 1.0825(5) 0.0342(6) 0.8193(3) 0.0364(18) Uani 1 1 d . . .
H4A H 1.1107 0.0621 0.7895 0.055 Uiso 1 1 calc R . .
H4B H 1.0917 -0.0370 0.8200 0.055 Uiso 1 1 calc R . .
H4C H 1.1153 0.0625 0.8527 0.055 Uiso 1 1 calc R . .
C5 C 0.8232(4) 0.0360(4) 0.9436(2) 0.0153(12) Uani 1 1 d . . .
C6 C 0.8514(5) -0.0665(5) 1.0209(2) 0.0289(15) Uani 1 1 d . . .
H6A H 0.8312 -0.1298 1.0339 0.043 Uiso 1 1 calc R . .
H6B H 0.8339 -0.0141 1.0440 0.043 Uiso 1 1 calc R . .
H6C H 0.9224 -0.0665 1.0213 0.043 Uiso 1 1 calc R . .
C7 C 0.2984(4) 0.2724(4) 0.8847(2) 0.0169(12) Uani 1 1 d . . .
C8 C 0.3898(4) 0.2742(4) 0.9158(2) 0.0166(12) Uani 1 1 d . . .
C9 C 0.4104(4) 0.2847(4) 0.9767(2) 0.0158(12) Uani 1 1 d . . .
C10 C 0.3962(5) 0.2040(5) 1.0594(2) 0.0288(15) Uani 1 1 d . . .
H10A H 0.3696 0.1437 1.0724 0.043 Uiso 1 1 calc R . .
H10B H 0.3647 0.2608 1.0731 0.043 Uiso 1 1 calc R . .
H10C H 0.4670 0.2068 1.0716 0.043 Uiso 1 1 calc R . .
C11 C 0.2090(4) 0.2990(4) 0.9091(2) 0.0174(12) Uani 1 1 d . . .
C12 C 0.0339(4) 0.2894(5) 0.9025(3) 0.0287(15) Uani 1 1 d . . .
H12A H -0.0234 0.2626 0.8795 0.043 Uiso 1 1 calc R . .
H12B H 0.0276 0.3608 0.9046 0.043 Uiso 1 1 calc R . .
H12C H 0.0381 0.2613 0.9382 0.043 Uiso 1 1 calc R . .
C13 C 0.2780(4) 0.7898(4) 0.9405(2) 0.0179(12) Uani 1 1 d . . .
C14 C 0.2615(5) 0.8350(4) 0.8892(2) 0.0223(13) Uani 1 1 d . . .
H14 H 0.2218 0.8039 0.8602 0.027 Uiso 1 1 calc R . .
C15 C 0.3054(5) 0.9268(5) 0.8821(3) 0.0250(14) Uani 1 1 d . . .
H15 H 0.2960 0.9566 0.8480 0.030 Uiso 1 1 calc R . .
C16 C 0.3631(5) 0.9746(4) 0.9253(3) 0.0246(14) Uani 1 1 d . . .
H16 H 0.3914 1.0366 0.9203 0.029 Uiso 1 1 calc R . .
C17 C 0.3783(5) 0.9297(4) 0.9757(2) 0.0230(14) Uani 1 1 d . . .
H17 H 0.4172 0.9618 1.0047 0.028 Uiso 1 1 calc R . .
C18 C 0.3364(4) 0.8377(4) 0.9838(2) 0.0189(13) Uani 1 1 d . . .
```

```
H18 H 0.3472 0.8079 1.0179 0.023 Uiso 1 1 calc R . .
C19 C 0.2615(4) 0.6198(4) 1.0147(2) 0.0187(12) Uani 1 1 d . . .
C20 C 0.3176(5) 0.5354(4) 1.0262(2) 0.0223(13) Uani 1 1 d . . .
H20 H 0.3433 0.5019 0.9987 0.027 Uiso 1 1 calc R . .
C21 C 0.3361(5) 0.4999(4) 1.0790(2) 0.0252(14) Uani 1 1 d . . .
H21 H 0.3736 0.4420 1.0868 0.030 Uiso 1 1 calc R . .
C22 C 0.2992(5) 0.5499(5) 1.1205(3) 0.0257(14) Uani 1 1 d . . .
H22 H 0.3117 0.5254 1.1559 0.031 Uiso 1 1 calc R . .
C23 C 0.2437(5) 0.6364(5) 1.1091(2) 0.0245(14) Uani 1 1 d . . .
H23 H 0.2197 0.6706 1.1370 0.029 Uiso 1 1 calc R . .
C24 C 0.2237(4) 0.6722(5) 1.0564(2) 0.0209(13) Uani 1 1 d . . .
H24 H 0.1860 0.7300 1.0486 0.025 Uiso 1 1 calc R . .
C25 C 0.0921(4) 0.6698(4) 0.9367(2) 0.0194(13) Uani 1 1 d . . .
C26 C 0.0366(4) 0.7579(4) 0.9288(2) 0.0205(13) Uani 1 1 d . . .
H26 H 0.0685 0.8187 0.9256 0.025 Uiso 1 1 calc R . .
C27 C -0.0663(5) 0.7541(5) 0.9257(3) 0.0271(14) Uani 1 1 d . . .
H27 H -0.1035 0.8127 0.9207 0.033 Uiso 1 1 calc R . .
C28 C -0.1141(5) 0.6636(5) 0.9299(3) 0.0297(15) Uani 1 1 d . . .
H28 H -0.1832 0.6617 0.9277 0.036 Uiso 1 1 calc R . .
C29 C -0.0592(5) 0.5753(5) 0.9376(3) 0.0281(15) Uani 1 1 d . . .
H29 H -0.0914 0.5144 0.9400 0.034 Uiso 1 1 calc R . .
C30 C 0.0427(5) 0.5791(5) 0.9416(3) 0.0253(14) Uani 1 1 d . . .
H30 H 0.0797 0.5204 0.9476 0.030 Uiso 1 1 calc R . .
C31 C 0.2669(5) 0.5916(4) 0.8957(2) 0.0210(13) Uani 1 1 d . . .
C32 C 0.2011(5) 0.5640(5) 0.8501(2) 0.0273(15) Uani 1 1 d . . .
H32 H 0.1332 0.5771 0.8483 0.033 Uiso 1 1 calc R . .
C33 C 0.2357(5) 0.5170(5) 0.8069(3) 0.0332(16) Uani 1 1 d . . .
H33 H 0.1912 0.4995 0.7761 0.040 Uiso 1 1 calc R . .
C34 C 0.3353(5) 0.4966(5) 0.8097(3) 0.0309(16) Uani 1 1 d . . .
H34 H 0.3583 0.4653 0.7806 0.037 Uiso 1 1 calc R . .
C35 C 0.4021(5) 0.5220(5) 0.8551(3) 0.0320(16) Uani 1 1 d . . .
H35 H 0.4694 0.5065 0.8570 0.038 Uiso 1 1 calc R . .
C36 C 0.3688(5) 0.5707(5) 0.8981(3) 0.0297(15) Uani 1 1 d . . .
H36 H 0.4139 0.5893 0.9284 0.036 Uiso 1 1 calc R . .
O9 O 0.0000 0.2682(9) 0.7500 0.114(4) Uani 1 2 d SD . .
C40 C -0.0073(16) 0.3703(12) 0.7734(7) 0.158(5) Uani 1 1 d DU . .
C37 C -0.0214(15) 0.4623(14) 0.7911(8) 0.158(5) Uani 1 1 d DU . .
O10 O 0.9357(18) 0.7523(17) 0.7728(10) 0.150(8) Uiso 0.50 1 d P . .
O11 O 0.3919(17) 0.3252(17) 0.1911(9) 0.139(7) Uiso 0.50 1 d P . .

loop_
 _atom_site_aniso_label
 _atom_site_aniso_U_11
 _atom_site_aniso_U_22
 _atom_site_aniso_U_33
 _atom_site_aniso_U_23
 _atom_site_aniso_U_13
 _atom_site_aniso_U_12
Fe1 0.0105(4) 0.0263(5) 0.0084(4) 0.0014(3) -0.0029(3) 0.0011(3)
Fe2 0.0106(4) 0.0253(5) 0.0085(4) -0.0010(3) -0.0030(3) 0.0008(3)
O1 0.016(2) 0.033(2) 0.025(2) 0.012(2) -0.0037(19) 0.0038(18)
O2 0.013(2) 0.073(3) 0.016(2) 0.016(2) -0.0007(18) 0.007(2)
O3 0.023(2) 0.026(2) 0.015(2) 0.0015(18) -0.0095(18) -0.0008(19)
O4 0.016(2) 0.029(2) 0.010(2) 0.0056(17) -0.0084(16) -0.0018(17)
```

O5 0.021(3) 0.042(3) 0.019(2) -0.012(2) 0.0043(19) -0.002(2)
O6 0.012(2) 0.038(2) 0.019(2) -0.0081(19) 0.0003(17) -0.0001(18)
O7 0.021(2) 0.029(2) 0.014(2) -0.0053(18) -0.0044(18) -0.0024(19)
O8 0.029(3) 0.029(2) 0.009(2) -0.0011(17) -0.0034(18) -0.0032(19)
S1 0.0122(8) 0.0422(9) 0.0091(7) 0.0036(6) -0.0017(6) 0.0045(6)
S2 0.0110(7) 0.0292(8) 0.0099(7) 0.0025(6) -0.0024(5) 0.0019(6)
S3 0.0123(7) 0.0261(7) 0.0096(7) -0.0001(6) -0.0031(5) -0.0017(6)
S4 0.0121(7) 0.0256(7) 0.0105(7) 0.0003(6) -0.0013(5) -0.0004(6)
S5 0.0120(7) 0.0309(8) 0.0097(7) -0.0026(6) -0.0030(6) 0.0018(6)
S6 0.0130(8) 0.0281(8) 0.0109(7) -0.0021(6) -0.0028(6) 0.0014(6)
P1 0.0157(8) 0.0208(7) 0.0147(8) -0.0014(6) -0.0042(6) -0.0008(6)
C1 0.009(3) 0.021(3) 0.017(3) 0.000(2) -0.006(2) 0.001(2)
C2 0.013(3) 0.018(3) 0.014(3) 0.003(2) -0.003(2) 0.000(2)
C3 0.016(3) 0.026(3) 0.014(3) -0.001(3) -0.001(2) 0.000(2)
C4 0.015(3) 0.073(5) 0.022(3) 0.010(3) 0.004(3) 0.012(3)
C5 0.013(3) 0.020(3) 0.012(3) 0.000(2) -0.001(2) 0.005(2)
C6 0.030(4) 0.040(4) 0.013(3) 0.012(3) -0.008(3) 0.000(3)
C7 0.018(3) 0.022(3) 0.010(3) 0.001(2) -0.001(2) 0.002(2)
C8 0.015(3) 0.023(3) 0.010(3) -0.004(2) -0.005(2) 0.001(2)
C9 0.011(3) 0.026(3) 0.010(3) 0.000(2) -0.001(2) 0.006(2)
C10 0.038(4) 0.034(4) 0.012(3) 0.000(3) -0.001(3) -0.003(3)
C11 0.017(3) 0.021(3) 0.012(3) -0.001(2) -0.004(2) -0.001(2)
C12 0.009(3) 0.044(4) 0.033(4) -0.005(3) 0.004(3) -0.001(3)
C13 0.016(3) 0.020(3) 0.018(3) 0.000(2) 0.003(2) 0.000(2)
C14 0.024(3) 0.028(3) 0.013(3) 0.002(2) -0.007(3) -0.004(3)
C15 0.021(3) 0.033(3) 0.021(3) 0.009(3) 0.002(3) 0.000(3)
C16 0.022(4) 0.022(3) 0.031(4) 0.003(3) 0.007(3) -0.004(3)
C17 0.021(3) 0.026(3) 0.021(3) -0.007(3) 0.001(3) -0.006(3)
C18 0.017(3) 0.027(3) 0.013(3) -0.002(2) 0.001(2) 0.005(3)
C19 0.014(3) 0.021(3) 0.018(3) -0.001(2) -0.006(2) -0.005(2)
C20 0.020(3) 0.023(3) 0.021(3) -0.006(3) -0.007(3) -0.001(3)
C21 0.026(4) 0.023(3) 0.023(3) 0.001(3) -0.008(3) -0.001(3)
C22 0.027(4) 0.027(3) 0.018(3) 0.006(3) -0.009(3) -0.006(3)
C23 0.023(4) 0.032(3) 0.018(3) -0.002(3) 0.002(3) -0.006(3)
C24 0.017(3) 0.027(3) 0.016(3) 0.002(3) -0.008(2) -0.001(3)
C25 0.016(3) 0.024(3) 0.015(3) -0.004(2) -0.006(2) 0.000(2)
C26 0.020(3) 0.022(3) 0.017(3) -0.005(2) -0.004(3) -0.006(3)
C27 0.019(3) 0.030(3) 0.032(4) -0.001(3) 0.002(3) 0.005(3)
C28 0.017(3) 0.036(4) 0.034(4) 0.001(3) -0.001(3) -0.005(3)
C29 0.022(4) 0.031(3) 0.029(4) 0.006(3) -0.001(3) -0.010(3)
C30 0.023(4) 0.025(3) 0.026(3) 0.000(3) -0.003(3) 0.001(3)
C31 0.022(3) 0.022(3) 0.018(3) -0.004(2) -0.002(3) -0.004(3)
C32 0.024(4) 0.034(4) 0.021(3) -0.006(3) -0.004(3) 0.000(3)
C33 0.036(4) 0.042(4) 0.020(3) -0.010(3) -0.002(3) 0.001(3)
C34 0.038(4) 0.034(4) 0.020(3) -0.004(3) 0.005(3) 0.003(3)
C35 0.018(4) 0.043(4) 0.033(4) -0.005(3) -0.003(3) 0.003(3)
C36 0.021(4) 0.043(4) 0.023(3) -0.009(3) -0.003(3) -0.001(3)
O9 0.152(13) 0.082(8) 0.091(9) 0.000 -0.033(8) 0.000
C40 0.157(11) 0.139(11) 0.156(12) -0.002(10) -0.043(10) -0.032(12)
C37 0.157(11) 0.139(11) 0.156(12) -0.002(10) -0.043(10) -0.032(12)

_geom_special_details
;
 All esds (except the esd in the dihedral angle between two l.s. planes)

```
 are estimated using the full covariance matrix.  The cell esds are taken
 into account individually in the estimation of esds in distances, angles
 and torsion angles; correlations between esds in cell parameters are only
 used when they are defined by crystal symmetry.  An approximate
(isotropic)
 treatment of cell esds is used for estimating esds involving l.s. planes.
;

loop_
 _geom_bond_atom_site_label_1
 _geom_bond_atom_site_label_2
 _geom_bond_distance
 _geom_bond_site_symmetry_2
 _geom_bond_publ_flag
Fe1 S3 2.1567(18) . ?
Fe1 S1 2.1827(18) . ?
Fe1 S2 2.2012(16) . ?
Fe1 S4 2.2443(17) . ?
Fe1 S4 2.2501(16) 4_657 ?
Fe1 Fe2 2.6952(13) . ?
Fe1 Fe2 2.7226(12) 4_657 ?
Fe2 S4 2.1574(18) . ?
Fe2 S6 2.1911(16) . ?
Fe2 S5 2.2018(17) . ?
Fe2 S3 2.2401(16) 4_657 ?
Fe2 S3 2.2588(17) . ?
Fe2 Fe1 2.7226(12) 4_657 ?
O1 C3 1.192(7) . ?
O2 C3 1.331(7) . ?
O2 C4 1.452(7) . ?
O3 C5 1.208(7) . ?
O4 C5 1.332(7) . ?
O4 C6 1.455(7) . ?
O5 C11 1.201(6) . ?
O6 C11 1.349(7) . ?
O6 C12 1.443(7) . ?
O7 C9 1.202(7) . ?
O8 C9 1.330(7) . ?
O8 C10 1.455(7) . ?
S1 C1 1.734(6) . ?
S2 C2 1.718(6) . ?
S3 Fe2 2.2401(16) 4_657 ?
S4 Fe1 2.2501(16) 4_657 ?
S5 C7 1.737(6) . ?
S6 C8 1.721(6) . ?
P1 C31 1.788(6) . ?
P1 C25 1.791(6) . ?
P1 C13 1.797(6) . ?
P1 C19 1.800(6) . ?
C1 C2 1.364(7) . ?
C1 C3 1.489(8) . ?
C2 C5 1.491(8) . ?
C4 H4A 0.9600 . ?
C4 H4B 0.9600 . ?
```

C4 H4C 0.9600 . ?
C6 H6A 0.9600 . ?
C6 H6B 0.9600 . ?
C6 H6C 0.9600 . ?
C7 C8 1.356(8) . ?
C7 C11 1.480(8) . ?
C8 C9 1.505(8) . ?
C10 H10A 0.9600 . ?
C10 H10B 0.9600 . ?
C10 H10C 0.9600 . ?
C12 H12A 0.9600 . ?
C12 H12B 0.9600 . ?
C12 H12C 0.9600 . ?
C13 C18 1.392(8) . ?
C13 C14 1.401(8) . ?
C14 C15 1.386(8) . ?
C14 H14 0.9300 . ?
C15 C16 1.384(9) . ?
C15 H15 0.9300 . ?
C16 C17 1.380(9) . ?
C16 H16 0.9300 . ?
C17 C18 1.383(8) . ?
C17 H17 0.9300 . ?
C18 H18 0.9300 . ?
C19 C20 1.367(8) . ?
C19 C24 1.412(8) . ?
C20 C21 1.386(9) . ?
C20 H20 0.9300 . ?
C21 C22 1.387(9) . ?
C21 H21 0.9300 . ?
C22 C23 1.386(9) . ?
C22 H22 0.9300 . ?
C23 C24 1.386(8) . ?
C23 H23 0.9300 . ?
C24 H24 0.9300 . ?
C25 C26 1.395(8) . ?
C25 C30 1.401(8) . ?
C26 C27 1.384(9) . ?
C26 H26 0.9300 . ?
C27 C28 1.385(9) . ?
C27 H27 0.9300 . ?
C28 C29 1.393(9) . ?
C28 H28 0.9300 . ?
C29 C30 1.369(9) . ?
C29 H29 0.9300 . ?
C30 H30 0.9300 . ?
C31 C32 1.381(9) . ?
C31 C36 1.400(9) . ?
C32 C33 1.391(9) . ?
C32 H32 0.9300 . ?
C33 C34 1.367(10) . ?
C33 H33 0.9300 . ?
C34 C35 1.379(10) . ?
C34 H34 0.9300 . ?

```
C35 C36 1.387(9) . ?
C35 H35 0.9300 . ?
C36 H36 0.9300 . ?
O9 C40 1.494(17) . ?
O9 C40 1.494(17) 4_557 ?
C40 C40 1.21(4) 4_557 ?
C40 C37 1.33(2) . ?

loop_
 _geom_angle_atom_site_label_1
 _geom_angle_atom_site_label_2
 _geom_angle_atom_site_label_3
 _geom_angle
 _geom_angle_site_symmetry_1
 _geom_angle_site_symmetry_3
 _geom_angle_publ_flag
S3 Fe1 S1 105.74(7) . . ?
S3 Fe1 S2 107.61(6) . . ?
S1 Fe1 S2 88.91(6) . . ?
S3 Fe1 S4 104.32(6) . . ?
S1 Fe1 S4 149.38(7) . . ?
S2 Fe1 S4 87.48(6) . . ?
S3 Fe1 S4 103.12(6) . 4_657 ?
S1 Fe1 S4 89.22(6) . 4_657 ?
S2 Fe1 S4 148.53(7) . 4_657 ?
S4 Fe1 S4 78.43(6) . 4_657 ?
S3 Fe1 Fe2 54.12(5) . . ?
S1 Fe1 Fe2 159.76(6) . . ?
S2 Fe1 Fe2 95.32(5) . . ?
S4 Fe1 Fe2 50.80(5) . . ?
S4 Fe1 Fe2 97.17(5) 4_657 . ?
S3 Fe1 Fe2 53.13(4) . 4_657 ?
S1 Fe1 Fe2 96.80(5) . 4_657 ?
S2 Fe1 Fe2 160.74(6) . 4_657 ?
S4 Fe1 Fe2 96.54(5) . 4_657 ?
S4 Fe1 Fe2 50.33(5) 4_657 4_657 ?
Fe2 Fe1 Fe2 73.27(4) . 4_657 ?
S4 Fe2 S6 108.31(6) . . ?
S4 Fe2 S5 104.68(6) . . ?
S6 Fe2 S5 89.00(6) . . ?
S4 Fe2 S3 103.43(6) . 4_657 ?
S6 Fe2 S3 147.23(7) . 4_657 ?
S5 Fe2 S3 90.74(6) . 4_657 ?
S4 Fe2 S3 103.81(6) . . ?
S6 Fe2 S3 87.07(6) . . ?
S5 Fe2 S3 151.02(7) . . ?
S3 Fe2 S3 77.57(7) 4_657 . ?
S4 Fe2 Fe1 53.72(5) . . ?
S6 Fe2 Fe1 95.56(5) . . ?
S5 Fe2 Fe1 158.25(6) . . ?
S3 Fe2 Fe1 96.66(5) 4_657 . ?
S3 Fe2 Fe1 50.68(5) . . ?
S4 Fe2 Fe1 53.40(4) . 4_657 ?
S6 Fe2 Fe1 161.63(6) . 4_657 ?
```

S5 Fe2 Fe1 97.21(5) . 4_657 ?
S3 Fe2 Fe1 50.38(5) 4_657 4_657 ?
S3 Fe2 Fe1 95.45(5) . 4_657 ?
Fe1 Fe2 Fe1 72.55(4) . 4_657 ?
C3 O2 C4 114.2(5) . . ?
C5 O4 C6 114.7(5) . . ?
C11 O6 C12 115.0(4) . . ?
C9 O8 C10 115.9(5) . . ?
C1 S1 Fe1 105.53(19) . . ?
C2 S2 Fe1 105.59(19) . . ?
Fe1 S3 Fe2 76.49(5) . 4_657 ?
Fe1 S3 Fe2 75.19(5) . . ?
Fe2 S3 Fe2 91.88(6) 4_657 . ?
Fe2 S4 Fe1 75.48(5) . . ?
Fe2 S4 Fe1 76.27(5) . 4_657 ?
Fe1 S4 Fe1 91.00(6) . 4_657 ?
C7 S5 Fe2 105.3(2) . . ?
C8 S6 Fe2 105.5(2) . . ?
C31 P1 C25 109.0(3) . . ?
C31 P1 C13 106.5(3) . . ?
C25 P1 C13 112.0(3) . . ?
C31 P1 C19 113.6(3) . . ?
C25 P1 C19 105.0(3) . . ?
C13 P1 C19 110.9(3) . . ?
C2 C1 C3 119.2(5) . . ?
C2 C1 S1 119.7(4) . . ?
C3 C1 S1 121.0(4) . . ?
C1 C2 C5 122.2(5) . . ?
C1 C2 S2 119.6(4) . . ?
C5 C2 S2 118.0(4) . . ?
O1 C3 O2 123.4(6) . . ?
O1 C3 C1 124.3(5) . . ?
O2 C3 C1 112.3(5) . . ?
O2 C4 H4A 109.5 . . ?
O2 C4 H4B 109.5 . . ?
H4A C4 H4B 109.5 . . ?
O2 C4 H4C 109.5 . . ?
H4A C4 H4C 109.5 . . ?
H4B C4 H4C 109.5 . . ?
O3 C5 O4 124.4(5) . . ?
O3 C5 C2 123.9(5) . . ?
O4 C5 C2 111.6(5) . . ?
O4 C6 H6A 109.5 . . ?
O4 C6 H6B 109.5 . . ?
H6A C6 H6B 109.5 . . ?
O4 C6 H6C 109.5 . . ?
H6A C6 H6C 109.5 . . ?
H6B C6 H6C 109.5 . . ?
C8 C7 C11 119.5(5) . . ?
C8 C7 S5 119.3(4) . . ?
C11 C7 S5 121.1(4) . . ?
C7 C8 C9 126.1(5) . . ?
C7 C8 S6 120.4(4) . . ?
C9 C8 S6 113.3(4) . . ?

O7 C9 O8 125.1(5) . . ?
O7 C9 C8 124.4(5) . . ?
O8 C9 C8 110.4(5) . . ?
O8 C10 H10A 109.5 . . ?
O8 C10 H10B 109.5 . . ?
H10A C10 H10B 109.5 . . ?
O8 C10 H10C 109.5 . . ?
H10A C10 H10C 109.5 . . ?
H10B C10 H10C 109.5 . . ?
O5 C11 O6 123.1(5) . . ?
O5 C11 C7 123.8(5) . . ?
O6 C11 C7 113.1(5) . . ?
O6 C12 H12A 109.5 . . ?
O6 C12 H12B 109.5 . . ?
H12A C12 H12B 109.5 . . ?
O6 C12 H12C 109.5 . . ?
H12A C12 H12C 109.5 . . ?
H12B C12 H12C 109.5 . . ?
C18 C13 C14 120.1(5) . . ?
C18 C13 P1 122.2(4) . . ?
C14 C13 P1 117.7(5) . . ?
C15 C14 C13 119.1(6) . . ?
C15 C14 H14 120.5 . . ?
C13 C14 H14 120.5 . . ?
C16 C15 C14 120.9(6) . . ?
C16 C15 H15 119.6 . . ?
C14 C15 H15 119.6 . . ?
C17 C16 C15 119.6(5) . . ?
C17 C16 H16 120.2 . . ?
C15 C16 H16 120.2 . . ?
C16 C17 C18 120.8(6) . . ?
C16 C17 H17 119.6 . . ?
C18 C17 H17 119.6 . . ?
C17 C18 C13 119.6(6) . . ?
C17 C18 H18 120.2 . . ?
C13 C18 H18 120.2 . . ?
C20 C19 C24 120.6(5) . . ?
C20 C19 P1 123.3(5) . . ?
C24 C19 P1 116.0(4) . . ?
C19 C20 C21 119.6(6) . . ?
C19 C20 H20 120.2 . . ?
C21 C20 H20 120.2 . . ?
C20 C21 C22 120.7(6) . . ?
C20 C21 H21 119.6 . . ?
C22 C21 H21 119.6 . . ?
C23 C22 C21 119.7(6) . . ?
C23 C22 H22 120.1 . . ?
C21 C22 H22 120.1 . . ?
C22 C23 C24 120.2(6) . . ?
C22 C23 H23 119.9 . . ?
C24 C23 H23 119.9 . . ?
C23 C24 C19 119.1(6) . . ?
C23 C24 H24 120.4 . . ?
C19 C24 H24 120.4 . . ?

```
C26 C25 C30 119.4(5) . . ?
C26 C25 P1 123.0(5) . . ?
C30 C25 P1 117.4(5) . . ?
C27 C26 C25 119.5(5) . . ?
C27 C26 H26 120.3 . . ?
C25 C26 H26 120.3 . . ?
C28 C27 C26 120.4(6) . . ?
C28 C27 H27 119.8 . . ?
C26 C27 H27 119.8 . . ?
C27 C28 C29 120.4(6) . . ?
C27 C28 H28 119.8 . . ?
C29 C28 H28 119.8 . . ?
C30 C29 C28 119.3(6) . . ?
C30 C29 H29 120.4 . . ?
C28 C29 H29 120.4 . . ?
C29 C30 C25 121.0(6) . . ?
C29 C30 H30 119.5 . . ?
C25 C30 H30 119.5 . . ?
C32 C31 C36 119.3(6) . . ?
C32 C31 P1 120.5(5) . . ?
C36 C31 P1 119.6(5) . . ?
C31 C32 C33 120.4(6) . . ?
C31 C32 H32 119.8 . . ?
C33 C32 H32 119.8 . . ?
C34 C33 C32 119.9(6) . . ?
C34 C33 H33 120.0 . . ?
C32 C33 H33 120.0 . . ?
C33 C34 C35 120.6(6) . . ?
C33 C34 H34 119.7 . . ?
C35 C34 H34 119.7 . . ?
C34 C35 C36 120.1(6) . . ?
C34 C35 H35 120.0 . . ?
C36 C35 H35 120.0 . . ?
C35 C36 C31 119.7(6) . . ?
C35 C36 H36 120.2 . . ?
C31 C36 H36 120.2 . . ?
C40 O9 C40 47.9(14) . 4_557 ?
C40 C40 C37 111.8(12) 4_557 . ?
C40 C40 O9 66.0(7) 4_557 . ?
C37 C40 O9 174.8(19) . . ?

_diffrn_measured_fraction_theta_max    0.998
_diffrn_reflns_theta_full              25.00
_diffrn_measured_fraction_theta_full   0.998
_refine_diff_density_max    1.927
_refine_diff_density_min   -0.809
_refine_diff_density_rms    0.159
```
